# Age-specific 1-year mortality rates after hip fracture based on the populations in mainland China between the years 2000 and 2018: a systematic analysis

**DOI:** 10.1007/s11657-019-0604-3

**Published:** 2019-05-25

**Authors:** Zhiyong Cui, Hui Feng, Xiangyu Meng, Siying Zhuang, Zhaorui Liu, Kaifeng Ye, Chuan Sun, Yong Xing, Fang Zhou, Yun Tian

**Affiliations:** 1 Department of Orthopedic Trauma, Peking University Third Hospital, No 49 Huayuan Road, Haidian District, Beijing, China; 2 Peking University Health Science Center, Beijing, China; 3 Department of Urology, Wuhan University Zhongnan Hospital, Wuhan, Hubei China; 4 Wuhan University School of Medicine, Wuhan, Hubei China; 5 Peking University Sixth Hospital, Beijing, China

**Keywords:** Hip fracture, Mortality, China, Systematic review, Meta-analysis

## Abstract

**Summary:**

We used statistical approaches to calculate 1-year mortality rates and reveal the relationship between age and the 1-year mortality rate after hip fracture based on data from mainland China between the years 2000 and 2018.

**Introduction:**

Data on the 1-year mortality rates after hip fracture in mainland China remain limited and localized. We aimed to analyze the 1-year mortality rates and reveal the variations in 1-year mortality by age after hip fracture based on data from mainland China.

**Methods:**

We searched PubMed, EMBASE, Cochrane Library, CNKI, Wanfang, and CBM-SinoMed for all relevant articles in English or Chinese to estimate the 1-year mortality rates after hip fracture in mainland China. A random-effects meta-analysis model was fitted to pool the overall 1-year mortality rates. A multilevel mixed-effects meta-regression model was developed. Based on the final model, the age-specific 1-year mortality rates after hip fracture in mainland China were generated.

**Results:**

The pooled estimate of the 1-year mortality rate was 13.96% after hip fracture (95% CI 12.26 to 15.86%), 17.47% after femoral intertrochanteric fracture (95% CI 14.29 to 21.20%), and 9.83% after femoral neck fracture (95% CI 6.96 to 13.72%) between the years 2000 and 2018. We found that the 1-year mortality rates ranged from 2.65% (95% CI 1.76 to 3.99%) in those aged 50~54 years to 28.91% (95% CI 24.23 to 34.30%) in those aged 95~99 years after hip fracture; ranged from 1.73% (95% CI 0.58 to 4.99%) in those aged 50~54 years to 50.11% (95% CI 46.03% to 53.97%) in those aged 95~99 years after femoral intertrochanteric fracture; and ranged from 1.66% (95% CI 1.31 to 2.11%) in those aged 60~64 years to 37.71% (95% CI 27.92 to 48.63%) in those aged 95~99 years after femoral neck fracture.

**Conclusion:**

In this systematic review and meta-analysis, we calculated the 1-year mortality rate after hip fracture in mainland China and found that this rate was lower than that in most countries. We also estimated the age-specific mortality rates for different age groups after hip fracture. These findings will be beneficial for the prevention and treatment of hip fracture in mainland China.

**Electronic supplementary material:**

The online version of this article (10.1007/s11657-019-0604-3) contains supplementary material, which is available to authorized users.

## Introduction

Hip fracture is one of the most devastating consequences of osteoporosis [1, 2] and is becoming one of the most important public health problems in the world [3]. Femoral intertrochanteric fracture and femoral neck fracture are the two main types. Hip fracture is regarded as a result of demographic aging, and age is the major risk factor for mortality in patients with hip fractures [4, 5]. Hip fractures can exact a terrible toll on the elderly and lead to severe complications and high mortality due to their severity and high economic cost [6, 7]. Therefore, many researchers have been paying increasing attention to mortality from hip fracture in the past two decades [8].

Data about the mortality risk and rates of hip fractures are available abroad and have contributed to the efforts and attention that national and local policymakers have devoted to improving patients’ healthcare quality and safety [9]. However, data about mortality rate after hip fracture in mainland China remain limited and localized. To our best knowledge, population-based studies on a national basis have never been conducted due to the difficulty of comprehensive inclusion of populations in a country of 1.3 billion people with different regional, racial, and ethnic groups, and the lack of these studies has hindered our understanding of the disease and adversely affected healthcare quality and safety. Fortunately, a growing number of population-based studies about the 1-year mortality rates after hip fracture were conducted in local areas in mainland China during the years 2000 and 2018. These studies were restricted to specific geographic and demographic features and could not represent the overall Chinese population. However, it is feasible to conduct a systematic synthesis of the data from population-based studies and explore the 1-year mortality rates after hip fracture from an epidemiological modeling approach.

In this study, we undertook a comprehensive systematic review and meta-analysis to analyze the 1-year mortality rates after hip fracture, femoral intertrochanteric fracture, and femoral neck fracture between years 2000 and 2018. We also aimed to develop epidemiological models to reveal the variations in 1-year mortality rate by age for hip fracture, femoral intertrochanteric fracture, and femoral neck fracture based on these data from mainland China.

## Methods

### Systematic review and meta-analysis

#### Search strategy

This systematic review and meta-analysis followed the guidelines of the Meta-analysis of Observational Studies in Epidemiology (MOOSE) [10]. We performed a systematic literature search for all relevant articles in English or Chinese in PubMed, EMBASE, Cochrane Library, China National Knowledge Infrastructure (CNKI), Wanfang, and Chinese Biomedicine Literature Database (CBM-SinoMed). We used Boolean operators to link Medical Subject Heading terms including *hip fracture*, *mortality*, and *China*. Specific search strategies were made that were adapted to fit features of the different bibliographic databases (see Table S1 in the Supplementary Material). We limited the publication years from 2000 to 2018. Additional search approaches were also used. We manually searched the unpublished conference reports and papers in the Peking University Health Science Library. The search occurred from December 1 to 31 in 2018, and the last search time was March 15, 2019. The search was conducted by two investigators in parallel; disagreements were discussed with a third investigator, who made the final decision.

#### Selection criteria

To be included in the systematic review and meta-analysis, studies needed to be population-based primary studies, reporting the 1-year mortality rates after hip fracture, femoral intertrochanteric fracture, or femoral neck fracture in populations with a defined age structure living in nursing homes or communities in mainland China. The diagnosis should be clearly stated. We only included studies with recruitment years ranging from 2000 to 2018. The data needed to be available and in a form that allowed for the calculation of 1-year mortality rates and reported the main characteristics of the study subjects. We excluded trials, cross-sectional studies, reviews, research letters, studies with less than 1-year of follow-up, studies with inconsistent results, and studies conducted in Taiwan, Hong Kong, Macao, or Chinese populations in other countries around the world. Duplicate articles were identified if they reported duplicate or overlapping results from the same study. We excluded the duplicate articles with the same results or included only the one with the most representative results. Pathological hip fracture was also excluded.

#### Quality assessment and data extraction

Two investigators used the Newcastle–Ottawa Scale (NOS) [11], which contains eight items, with one or two scores for each item, to assess the quality of the cohort studies. The studies with zero to three points were excluded because they were considered to have a high risk of bias. We read the articles that met the selection criteria in detail and extracted customized data information from the included studies. For the purpose of this study, hip fracture was classified into two main types: femoral intertrochanteric fracture and femoral neck fracture. Subtrochanteric fractures were not included because of the limited data availability. Relevant data on hip fracture and the two subtypes were separately extracted from the studies. The study regions were classified into six geographic regions according to the definitions of the National Bureau of Statistics: East China, North China, Northeast China, Northwest China, South Central China, and Southwest China. The data extraction included the study’s main characteristics (first author’s name, the publication date, the survey year, the follow-up time, the study types, the settings and the geographic regions of the study area, etc.), the age (mean or median age, or midpoint of the age range) of the populations, 1-year mortality cases and sample numbers by the fracture types, age groups, and geographic locations. The extraction process was also completed by two investigators independently.

### Statistical analysis and epidemiological model construction

In this study, we used random-effects models (the DerSimonian and Laird method) to calculate the overall 1-year mortality rates and 95% confidence intervals (CIs) for the populations with hip fracture, femoral intertrochanteric fracture, and neck fracture between the years 2000 and 2018, because significant heterogeneity was found across studies. Statistical heterogeneity was quantified with Cochrane’s *Q* statistic and *I*^2^ metrics. The *I*^2^ value lies between 0 and 100%. A value of 0% indicates no observed heterogeneity and larger values show increasing heterogeneity. A *p* value < 0.05 of the Cochrane’s *Q* statistic indicates statistically significant between-study heterogeneity. We drew funnel plots to address publication bias visually and conducted Egger’s linear correlation test to identify any publication bias. We also used the trim and fill method to provide a summary effect adjusted for publication bias. Additionally, leave-one-out sensitivity analysis was performed to assess the stability of the pooled results.

Moreover, we used multilevel meta-regression to model the 1-year mortality of disease in the population, which has been used widely in assessing the prevalence of diseases [12, 13] but has never been used for mortality. The logit transformation of the mortality data was adopted in the regression. We developed three meta-regression models about 1-year mortality rates for hip fracture, femoral intertrochanteric fracture, and neck fracture. The three models were based on 114 data points from 54 studies; 75 data points for hip fracture, 27 data points for femoral intertrochanteric fracture, and 12 data points for femoral neck fracture. We used a multilevel mixed-effects meta-regression model for hip fracture, femoral intertrochanteric fracture, and femoral neck fracture because the same study often offered different data points in the analysis.

The association of the mortality rate and all of the individual variables, such as age, setting (urban, rural, and mixed), geographic region (north, northeast, east, south central, southwest, northwest), study type (prospective or retrospective cohort study), and survey year, was explored with univariable meta-regression for hip fracture, femoral intertrochanteric fracture, and neck fracture, respectively. Age was found to be the only factor that was significantly associated with the 1-year mortality rate after the fractures (Table S2 in the Supplementary Material). The final meta-regression was constructed by taking the variation of age. We used the binomial distribution to model mortality and took into account the sample size of the studies. Given that

$$ 1-\mathrm{yearmortalityrate}=\mathrm{P}=\frac{1-\mathrm{yearmortalitycases}}{\mathrm{numberofthetotalpatients}} $$

then we used the logit transformation:

$$ \mathrm{logit}\left(\mathrm{P}\right)=\ln \left(\frac{P}{1-P}\right)=\upalpha +{\upbeta}_1\ast {\mathrm{X}}_1+{\upbeta}_2\ast {\mathrm{X}}_2+\dots $$

age is considered as the only factor associated with the fractures

$$ \ln \left(\frac{P}{1-P}\right)=\upalpha +\upbeta \ast \left(\mathrm{age}\right) $$

thus,

$$ \frac{P}{1-P}={\mathrm{e}}^{\upalpha +\upbeta \ast \left(\mathrm{age}\right)} $$

and

$$ 1-\mathrm{yearmortalityrate}=\mathrm{P}=\frac{{\mathrm{e}}^{\upalpha +\upbeta \ast \left(\mathrm{age}\right)}}{1+{\mathrm{e}}^{\upalpha +\upbeta \ast \left(\mathrm{age}\right)}} $$

Therefore, we estimated the age-specific mortality rates for the median year of every 5-year age group following the model above. The midpoint of the age range was adopted as the age variable for analysis. The age ranged from 50 to 100 years for hip fracture and intertrochanteric fracture, and from 60 to 100 years for femoral neck fracture because no data points were available to construct the regression model for femoral neck fracture when the age was younger than 60 years. For studies with censoring age groups, e.g., older than 90 years, the missing age band was taken as the same width as other age groups in the same paper. All of the statistical analyses were performed using R software, version 3.5.1 (R Foundation for Statistical Computing, Vienna, Austria). A two-tailed value of *P* < 0.05 was considered statistically significant.

## Results

### Literature characteristics

**Fig. 1:**
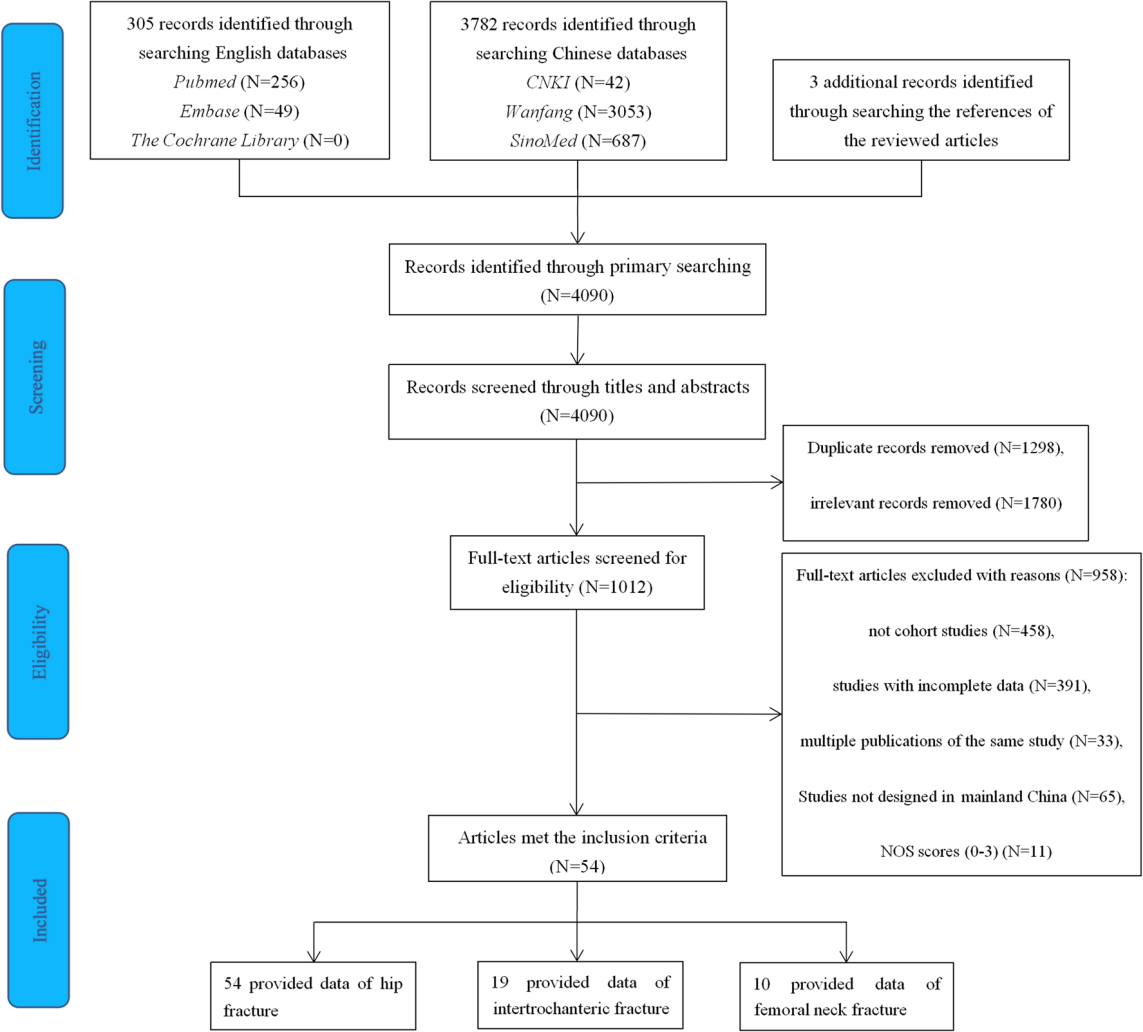
Flowchart of selection of cohort studies of 1-year mortality after hip fracture in mainland China following the MOOSE guideline

**Fig. 2:**
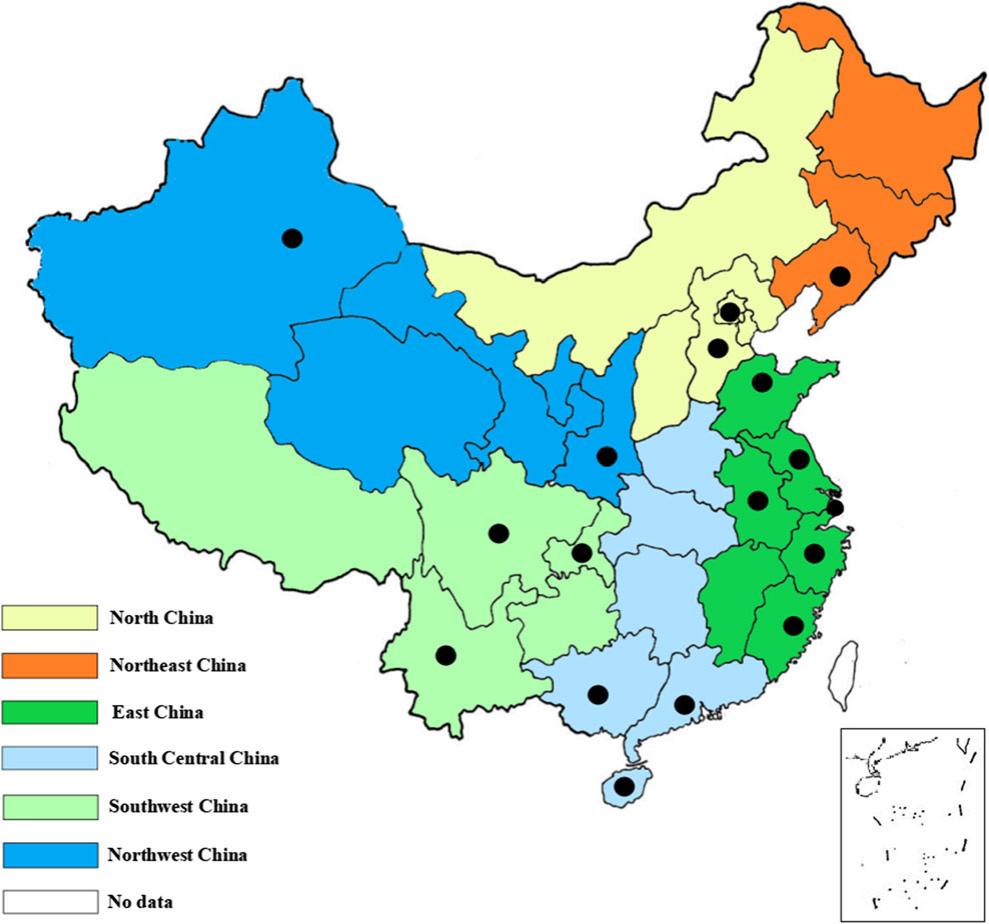
A map of study locations included in the systematic review in mainland China. Dots indicate the study locations

We initially retrieved 4090 records from the primary searches and obtained additional three records from searching the references of the reviewed articles. A total of 305 records were from PubMed, EMBASE, and the Cochrane Library, and 3782 publications were from the CNKI, Wanfang, and CBM-SinoMed. After scanning the titles and abstracts, we reviewed 1012 papers in full and 54 studies ultimately met the selection criteria. A full list of the included studies is shown in Table S3 in the Supplementary Material. The selection process and the reasons are described in detail in the flowchart in Fig. 1. The 54 studies included in this meta-analysis involved 22,817 Chinese individuals in 13 provinces, 3 municipalities, and 1 autonomous region, covering all of the six regions in mainland China (Fig. 2). There were 49 retrospective cohort studies and 5 prospective cohort studies, and 54 studies focused on hip fractures, 19 studies reported on femoral intertrochanteric fractures, and 10 were about femoral neck fractures. The majority of the studies (66.7%, *N* = 36) were conducted in urban areas. The main and detailed characteristics of the studies are listed in Table 1 and Table S4 in the Supplementary Material. The recruitment time of the 54 cohort studies covered from 2000 to 2015, and the publication time was from 2007 to 2018. The mean ages of the subjects ranged from 70 to 93.7, and the 1-year mortality rates after hip fracture ranged from 1.53 to 33.86%.

**Table 1:** The main characteristics of 54 studies included in this systematic review

Characteristics of studies	Numbers of studies (%)
Initial time of the study	
2000–2009	30 (55.6)
2010–2018	24 (44.4)
Setting	
Urban	36 (66.7)
Rural	2 (3.7)
Mixed	16 (29.6)
Sample size	
≤ 500	44 (81.5)
500–1000	4 (7.5)
1000–1500	3 (5.5)
> 1500	3 (5.5)
Geographic regions	
North China	14 (25.9)
Northeast China	1 (1.9)
East China	24 (44.4)
South Central China	7 (13.0)
Southwest China	6 (11.1)
Northwest China	2 (3.7)
Fracture types	
Femoral intertrochanteric fracture	19 (35.2)
Femoral neck fracture	10 (18.5)
Hip fracture	54 (100.0)
Study types	
Prospective cohort study	5 (9.3)
Retrospective cohort study	49 (90.7)

### Methodological quality

We used the NOS scores to evaluate the methodological quality of the 54 studies. NOS scores of the studies ranged from 4 to 7, and the average was 5.07. Eight studies were scored 7, which was considered high quality. The NOS scores of each study are shown in Table S5 in the Supplementary Material.

### Meta-analysis and meta-regression of the 1-year mortality rates

Meta-analysis of the reported 1-year mortality revealed significantly high heterogeneity among studies of hip fracture (*I*^2^ = 93%, *p* < 0.01), femoral intertrochanteric fracture (*I*^2^ = 90%, *p* < 0.01), and femoral neck fracture (*I*^2^ = 76%, *p* < 0.01). A random-effects model was used. The pooled estimates of the 1-year mortality rate were 13.96% after hip fracture (95% CI 12.26 to 15.86%, Fig. S1 in the Supplementary Material), 17.47% after femoral intertrochanteric fracture (95% CI 14.29 to 21.20%, Fig. S2 in the Supplementary Material), and 9.83% after femoral neck fracture (95% CI 6.96 to 13.72%, Fig. S3 in the Supplementary Material).

Only age was revealed as a significant factor for the 1-year mortality rate after hip fracture, femoral intertrochanteric fracture, and femoral neck fracture in the univariable meta-regression analyses. The final meta-regression was constructed by taking the variation of age, and the results are shown in Table S2 in the Supplementary Material.

### Publication bias and sensitivity analysis

We used a funnel plot and Egger’s test to identify the publication bias in the results. For intertrochanteric fracture and neck fracture, Egger’s test (Fig. S4 and Fig. S5 in the Supplementary Material) indicated that there was no significant publication bias (*p* = 0.077 and *p* = 0.331, respectively). However, we observed publication bias in hip fracture according to the funnel plot and Egger’s test (*p* < 0.05) (Fig. S6 in the Supplementary Material). Then, we performed a trim and fill method to address the problem of publication bias (Fig. S6 in the Supplementary Material). We found the result changed a little after applying the trim and fill method.

For the sensitivity analysis, we used leave-one-out sensitivity analysis to remove one single study from the overall pooled analysis each time to expose the influence the removed data had on the overall results. We found that no single study significantly influenced the reliability and stability of the 1-year mortality rates (Fig. S7–S9 and Table S6–S8 in the Supplementary Material).

### Age-specific mortality rates after hip fracture, intertrochanteric fracture, or neck fracture

**Fig. 3:**
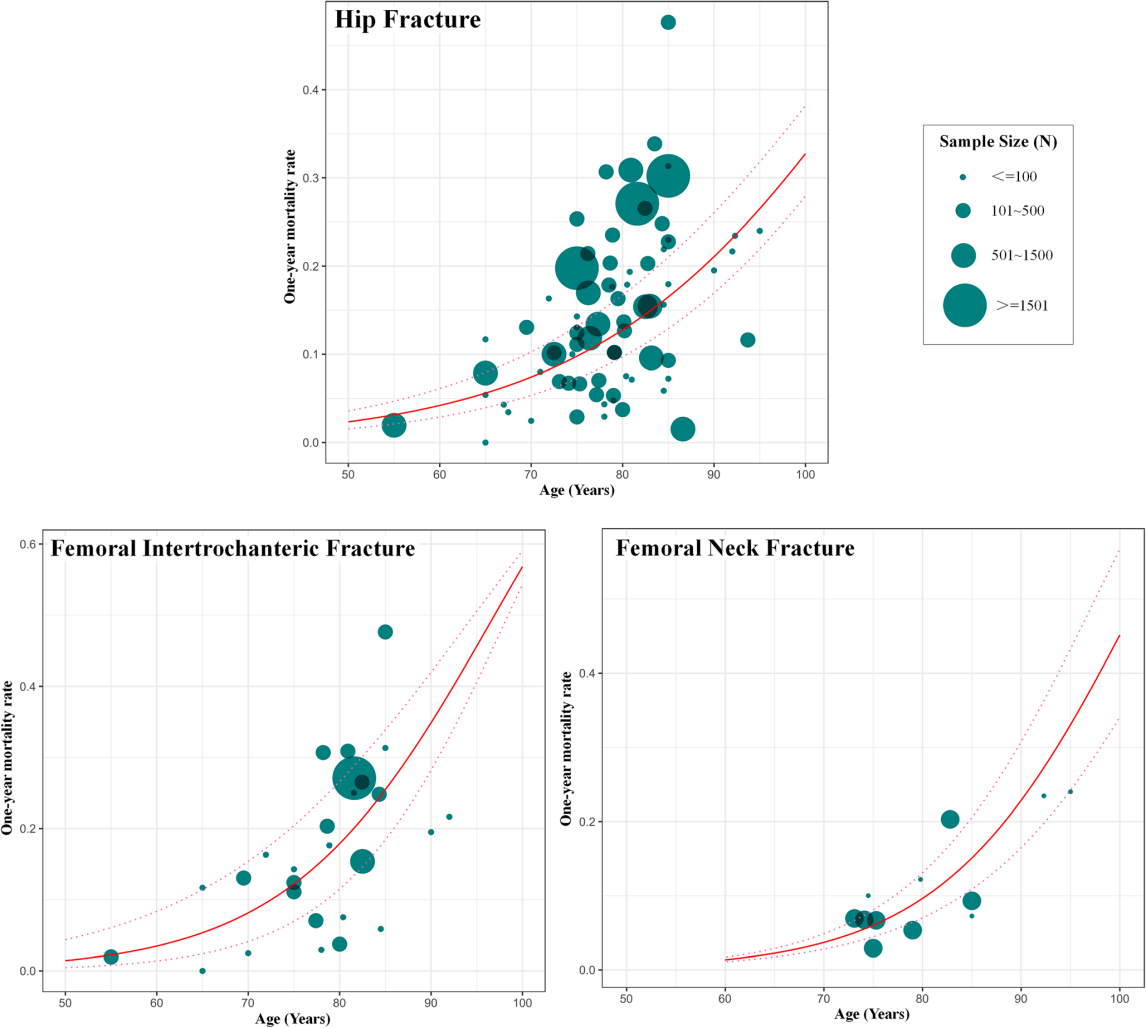
Age-specific 1-year mortality after hip fracture, femoral intertrochanteric fracture, and femoral neck fracture based on the data points from the included studies, with 95% confidence intervals. Note: The size of each bubble is proportional to the sample size. Regression lines are based on only or very few data points at younger (< 70 years) and older (> 90 years) ages. Overall, there were 75 data points for constructing the relationship between age and 1-year mortality after hip fracture, 27 data points for femoral intertrochanteric fracture, and 12 data points for femoral neck fracture

**Fig. 4:**
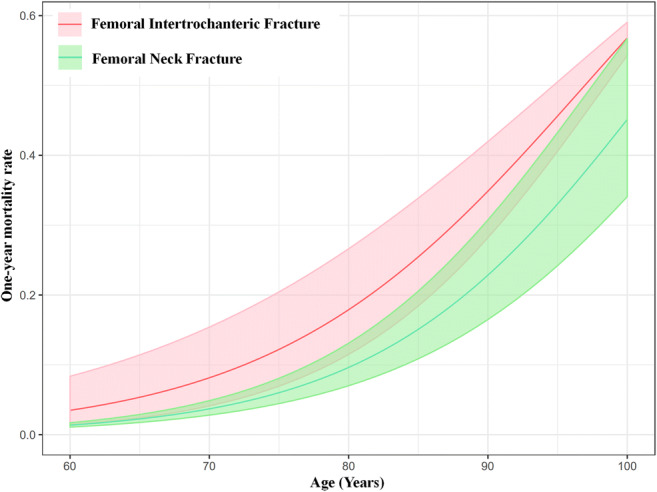
Comparison of estimated age-specific 1-year mortality rates between femoral intertrochanteric fracture and femoral neck fracture, with 95% confidence intervals

We constructed the relationship between ages and 1-year mortality rates based on the substantial number of data points from the included studies (Fig. 3). The age range was set as from 50 to 100 years for hip fracture and intertrochanteric fracture and from 60 to 100 years for femoral neck fracture, because no data points were available at younger ages (50~60 years old) for femoral neck fracture. The estimated age-specific 1-year mortality rates after hip fracture, femoral intertrochanteric fracture, and femoral neck fracture are shown in Table 2. An advanced age was revealed as the significant risk factor for the mortality rate and was more pronounced for femoral intertrochanteric fracture (Fig. 4). The 1-year mortality rates after the hip fracture ranged from 2.65% (95% CI 1.76 to 3.99%) in people aged 50~54 years to 28.91% (95% CI 24.23 to 34.30%) in people aged 95~99 years. The 1-year mortality rates after the femoral intertrochanteric fracture ranged from 1.73% (95% CI 0.58 to 4.99%) in people aged 50~54 years to 50.11% (95% CI 46.03 to 53.97%) in people aged 95~99 years. The 1-year mortality rates after the femoral neck fracture ranged from 1.66% (95% CI 1.31 to 2.11%) in people aged 60~64 years to 37.71% (95% CI 27.92 to 48.63%) in people aged 95~99 years.

**Table 2:** Estimated age-specific 1-year mortality rates after hip fracture, femoral intertrochanteric fracture, and femoral neck fracture

Age (years)	One-year mortality rate after hip fracture (95% CIs)	One-year mortality rate after femoral intertrochanteric fracture (95% CIs)	One-year mortality rate after femoral neck fracture (95% CIs)
50~54	0.0265	0.0173	–
	0.0176~0.0399	0.0058~0.0499	–
55~59	0.0354	0.0268	–
	0.0240~0.0523	0.0100~0.0691	–
60~64	0.0473	0.0414	0.0166
	0.0328~0.0681	0.0174~0.0948	0.0131~0.0211
65~69	0.0628	0.0634	0.0274
	0.0446~0.0882	0.0299~0.1289	0.0211~0.0356
70~74	0.0830	0.0960	0.0449
	0.0605~0.1135	0.0508~0.1728	0.0337~0.0597
75~79	0.1089	0.1427	0.0727
	0.0815~0.1450	0.0852~0.2278	0.0534~0.0983
80~84	0.1417	0.2069	0.1156
	0.1089~0.1835	0.1395~0.2940	0.0837~0.1576
85~89	0.1823	0.2902	0.1789
	0.1441~0.2293	0.2199~0.3703	0.1288~0.2431
90~94	0.2314	0.3906	0.2665
	0.1883~0.2827	0.3290~0.4536	0.1931~0.3554
95~99	0.2891	0.5011	0.3771
	0.2423~0.3430	0.4603~0.5397	0.2792~0.4863

*–* data was not available, *CIs* confidence intervals

The results are estimated predictions and are outside the range of the original data for hip fracture at the ages 50~54 years and 95~99 years, for femoral intertrochanteric fracture at the ages 50~54 years and 95~99 years, for femoral neck fracture at the ages 60~64 years, 65~69 years and 95~99 years

## Discussion

It is widely known that hip fracture is one of the most relevant fractures in terms of its high mortality [7]. It has been reported that having a hip fracture can cause excess mortality and that there is a greater mortality risk for patients with hip fracture than for those without hip fracture [6, 14]. There have been different results reported for the 1-year mortality rates after hip fracture. Haleem et al. [15] reported that the 1-year mortality rates were 22 to 29% from 1996 to 1998, while one single center survey [16] noted a 1-year mortality rate of 40% in American nursing home residents between 1999 and 2006. Abrahamsen [17] and Hu [18] performed meta-analyses and found that the mortality rates at 1 year were 5.9~59% and 24.5%, respectively. The 1-year mortality rate after hip fracture in Canadian nursing home residents was reported at approximately 45% in 2008 and 2009 [19]. In Asian populations, the 1-year mortality rates were also different: 17.8% in Korean women [20], 18.65% in Hong Kong [21], and 13.5% in Taiwanese women [22]. In mainland China, the local survey rate [23] regarding 1-year mortality in Beijing was approximately 23.44% in total. However, the result in Beijing could not represent the overall mortality in mainland China due to the unbalanced health care services between areas.

We performed this meta-analysis and systematic review based on the rigorous reviews of existing evidence on hip fracture mortality in mainland China. We calculated the 1-year mortality rates after hip fracture, femoral intertrochanteric fracture, and neck fracture in general Chinese populations between the years 2000 and 2018 and explored the variations with the age factors. The 1-year mortality rate after intertrochanteric fractures was higher than the femoral neck fracture (17.47% vs 9.83%), and the overall 1-year mortality rate after hip fracture was 13.96% in mainland China. Our study also estimated the age-specific mortality rates for the median year of every 5-year age group and revealed that advanced age was a significant risk factor for mortality. The 1-year mortality rate after hip fracture was the highest at 28.91% in the age group 95 to 99 years old and the lowest at 2.65% in the age group 50 to 54 years old. The strong association between the mortality rate and the age was consistent with other studies [6, 14]. Keene et al. [24] proposed that 1-year mortality would increase by 1% with a 1-year increase in age.

We also found that the 1-year mortality rate was higher after femoral intertrochanteric fracture than femoral neck fracture in every 5-year age group when the age was over 60 years old. The relationship between fracture type and mortality remains controversial, with some investigators [25] reporting that the fracture type did not affect the mortality while some survival studies [26, 27] revealed the mortality rates of intertrochanteric fracture patients were higher than femoral neck fracture patients. However, patients with intertrochanteric fractures were older than those with a femoral neck fracture in the previous studies. Whether the excess mortality in these studies reflects differences in age or the fracture types remains to be clarified. In our study, we compared the 1-year mortality rates between the femoral intertrochanteric fracture and neck fracture in the same age groups and found that the mortality rates were higher in those with intertrochanteric fracture, which suggested that fracture type might be one predictor of mortality in hip fracture patients. However, we did not compare the differences of the sex, comorbidities, anesthesia type, or other factors between the two fracture types in the same age group. These factors can also contribute to mortality after a hip fracture [14, 44].

The 1-year mortality rates in mainland China were lower than that in Canada, the USA, and most other regions in Asia mentioned above [19–22]. Different countries and races have different nutritional habits [23] and different physical exercise habits [28], which could cause differences in mortality. In addition, we should also take ethnic genetic variations into consideration. Some studies [29, 30] have demonstrated a relationship between bone mass and ethnic genes, showing that on average blacks have greater bone mass than whites and that Asians have the lowest bone mass among these races. Walker and colleagues [31] also indicated genetic differences in osseous microarchitecture in Chinese–American patients. However, there are few studies about the influence of special ethnicity on prognosis differences after hip fracture [32]. Although some studies [33, 34] have shown that black patients are at greater risk for mortality, others [35] did not reach the same conclusions. Therefore, it remains unclear whether the genetic variations between Chinese populations and others could affect the mortality rate after hip fracture.

In addition to the ethnic genetic variations, we should pay more attention to the impact of cultural and economic differences between different ethnic groups on mortality after hip fracture. The fact that Chinese cultural attitudes regarding hip fracture is different from others is manifested in several respects. On the one hand, the attitude towards the disease reflects cultural differences. Attitudes towards hip fracture might prolong the wait time for surgery, which has been demonstrated to be associated with a poor prognosis after hip fracture [36]. The delayed phenomenon is common in mainland China due to geographic and economic factors and may play an important role in mortality rates. On the other hand, we hypothesize that the reason the mortality rates were lower in mainland China than in the USA results from the higher mortality rates in nursing home residents [37, 38]. Elderly Chinese people are unwilling to separate from their children and to live in nursing homes because of their traditional cultural attitudes and the low quality of care in nursing homes. Consequently, community dwellers make up a large part of the elderly Chinese people, whose fracture rates are lower than that of nursing home residents [37, 38]. Therefore, different proportions of nursing home residents would lead to different mortality rates in different races and nations.

The economy undoubtedly has an important impact on the prognosis of diseases. Economic pressure poses challenges for China and other low-income countries in improving the medical resources and care quality [39]. It has been proposed that patients from economically disadvantaged areas are predisposed to delayed surgery for hip fracture, which would increase the mortality rates [37]. China has been undergoing rapid economic development in the past two decades [39]. The annual disposable income in urban and rural areas in 2008 was 3.0 and 2.2 times higher than that in 1998, respectively [39, 40], which results in a decrease in the morbidity and mortality of diseases. Nevertheless, it is still under debate whether socioeconomic status has an impact on mortality after hip fracture. Studies from the UK noted that lower socioeconomic status was relevant to higher mortality risk after hip fracture [41, 42], while investigators in the USA disagreed with these viewpoints [43].

To our knowledge, this is the first review to provide complete data regarding hip fracture mortality rates in mainland China. We conducted a comprehensive literature search and used a strict approach to include studies in order to reasonably cover the Chinese population in mainland China. The information bias due to selection and methodological heterogeneity was reduced to the minimum. The estimated results were representative for mainland China with a wide geographical scope covering all six geographic regions of China. We also constructed the epidemiological model to reveal the relationship between the age and mortality. This is the first time anyone has applied this epidemiological model to assessing the mortality of diseases. We found that age was a significant risk factor for the mortality rate, and we found that the mortality rates after femoral intertrochanteric fracture were higher than femoral neck fracture when the age group was the same. From a public health management perspective, these data can help to identify the prognosis after hip fracture in mainland China and help policy makers to allocate medical resources appropriately for hip fracture.

Our study is still subject to some potential limitations. First, heterogeneity was significant among the included studies although we used a rigorous selection approach. To explain the significant heterogeneity, we conducted meta-regression to examine the group-level variables, age, setting (urban, rural, and mixed), geographic region, study type (prospective or retrospective cohort study), and survey year, but could not explore the impact of individual-level variables because of the lack of information, for example, in regard to the sex [14], comorbid conditions [44], wait time for surgery [36], postsurgery complications [37, 45], advanced cognitive impairment [45], and increased baseline ADL (activities of daily living) [45]. The varied quality of the studies involved could also contribute to the heterogeneity. Second, publication bias was observed when analyzing the studies about hip fracture although the estimated results changed only a little after applying the trim and fill method. On the one hand, the publication bias might derive from the significant heterogeneity between the included studies. On the other hand, we could not avoid the absence of unpublished studies, which might lead to bias in the final estimates. Third, although 22,817 Chinese individuals were involved in our study, this number might be inadequate for one country with a population of 1.3 billion people. We did not include certain provinces, municipalities and autonomous regions such as Qinghai Province and the Inner Mongolia autonomous region. The mortality in these regions might have an impact on the results. Fourth, as mentioned above, there is a difference in mortality between nursing home residents and community dwellers. However, the studies included in the meta-analysis did not differentiate between nursing home residents and community dwellers, which could overestimate the mortality rates for community dwellers. Fifth, the studies included were based on patients admitted to the hospital, which would lead to missing those who were not admitted to hospitals with hip fracture and would result in selection bias.

## Conclusion

The 1-year mortality rate after hip fracture in mainland China between the years 2000 and 2018 was 13.96%, while for intertrochanteric fractures, it was 17.47%, and for femoral neck fracture, it was 9.83%. We also estimated the age-specific mortality rates for the median year of every 5-year age group and found that advanced age was a significant risk for the 1-year mortality rate after hip fracture, femoral intertrochanteric fracture, and femoral neck fracture. These findings will be beneficial for the prevention and treatment of hip fracture in mainland China. We also hope more elaborate studies will be conducted in future to analyze the mortality rates after hip fracture in mainland China.

## Electronic supplementary material

Table S1 (DOCX 16 kb)

Table S2 (DOCX 14 kb)

Table S3 (DOCX 27 kb)

Table S4 (DOCX 29 kb)

Table S5 (DOCX 24 kb)

Table S6 (DOCX 21 kb)

Table S7 (DOCX 15 kb)

Table S8 (DOCX 14 kb)

Figure S1 Forest plot of the one-year mortality rate after hip fracture in mainland China. A total of 54 studies were included in the meta-analysis. The one-year mortality rate was calculated as 13.96% (95% CI 12.26% to 15.86%) using a random-effects model. (PNG 1697 kb)

High resolution image (TIF 7575 kb)

Figure S2 Forest plot of the one-year mortality rate after femoral intertrochanteric fracture in mainland China. A total of 19 studies were included in the meta-analysis. The one-year mortality rate was calculated as 17.47% (95% CI 14.29% to 21.20%) using a random-effects model. (PNG 697 kb)

High resolution image (TIF 3096 kb)

Figure S3 Forest plot of the one-year mortality rate after femoral neck fracture in mainland China. A total of 10 studies were included in the meta-analysis. The one-year mortality rate was calculated as 9.83% (95% CI 6.96% to 13.72%) using a random-effects mode. (PNG 450 kb)

High resolution image (TIF 1998 kb)

Figure S4 Publication bias of one-year mortality after femoral intertrochanteric fracture. Note: (A) Funnel plot, (B) Egger’s test. (PNG 288 kb)

High resolution image (TIF 3176 kb)

Figure S5 Publication bias of one-year mortality after femoral neck fracture. Note: (A) Funnel plot, (B) Egger’s test. (PNG 239 kb)

High resolution image (TIF 2867 kb)

Figure S6 Publication bias of one-year mortality after hip fracture. Note: (A) Funnel plot, (B) Egger’s test, (C) Funnel plot after the trim and fill method. (PNG 535 kb)

High resolution image (TIF 5407 kb)

Figure S7 Leave-one-out sensitivity analysis of one-year mortality after hip fracture. (PNG 1464 kb)

High resolution image (TIF 7015 kb)

Figure S8 Leave-one-out sensitivity analysis of one-year mortality after femoral intertrochanteric fracture. (PNG 788 kb)

High resolution image (TIF 3895 kb)

Figure S9 Leave-one-out sensitivity analysis of one-year mortality after femoral neck fracture. (PNG 480 kb)

High resolution image (TIF 2364 kb)
