# Supplementary material for: Age-specific 1-year mortality rates after hip fracture based on the populations in mainland China between the years 2000 and 2018: a systematic analysis
Source: Arch Osteoporos. 2019 May 25;14(1):55. doi: 10.1007/s11657-019-0604-3 (PMC6535151; doi:10.1007/s11657-019-0604-3)
Supplement: Supplementary file 1 — (DOCX 16 kb) [file 11657_2019_604_MOESM1_ESM.docx]

| **CNKI**  Search Strategy:  (SU=’髋部骨折’+’转子间骨折’+’粗隆间骨折’+’股骨颈骨折’+’转子下骨折’) AND (SU=’死亡率’+’病死率’+’生存率’+’死亡’+’病死’+’生存’) AND (SU=’1年’+’12月’)  Access Date: from 01/12/2018 to 31/12/2018  Published time: from 01/01/2000 to 30/11/2018  **Wanfang**  Search Strategy  (主题:(髋部骨折)+主题:(转子间骨折)+主题:(粗隆间骨折)+主题:(股骨颈骨折)+主题:(转子下骨折)) AND (主题:(死亡率)+主题:(病死率)+主题:(生存率)+主题:(死亡)+主题:(病死)+主题:(生存)) AND (主题:(1年)+主题:(12月))  Access Date: from 01/12/2018 to 31/12/2018  Published time: from 2000 to 2018  **SinoMed**  Search Strategy  (髋部骨折 OR 转子间骨折 OR 粗隆间骨折 OR 股骨颈骨折 OR 转子下骨折) AND ( 死亡率 OR 病死率OR 生存率 OR 死亡 OR 病死 OR 生存) AND (1年OR 12月)  Access Date: from 01/12/2018 to 31/12/2018  Published time: from 2000 to 2018  **Pubmed**  Search Strategy  #1 Mortality[MeSH Terms] OR Mortality OR Fatality OR Death OR Survive  #2 China[MeSH Terms] OR China OR People's Republic of China OR mainland China  #3 hip fracture[MeSH Terms] OR hip fracture OR Fractures, Hip OR Trochanteric Fractures OR Fractures, Trochanteric OR Intertrochanteric Fractures OR Fractures, Intertrochanteric OR Subtrochanteric Fractures OR Fractures, Subtrochanteric OR Femoral Neck Fracture OR Femur Neck Fractures OR Femur Neck Fracture  #4 one year OR twelve months OR 365 days  #5 #1 AND #2 AND #3 AND #4  Access Date: from 01/12/2018 to 31/12/2018  Published time: from 01/01/2000 to 30/11/2018  **EMBASE**  Search Strategy  #1 ’hip fracture’/exp OR ’hip fracture’  #2 ’acetabulum fracture’/exp OR ’acetabulum fracture’  #3 ’femoral head fracture’/exp OR ’femoral head fracture’  #4 ’femoral neck fracture’/exp OR ’femoral neck fracture’  #5 ’femur intertrochanteric fracture’/exp OR ’femur intertrochanteric fracture’  #6 ’femur pertrochanteric fracture’/exp OR ’femur pertrochanteric fracture’  #7 ’femur subtrochanteric fracture’/exp OR ’femur subtrochanteric fracture’  #8 ’femur trochanteric fracture’/exp OR ’femur trochanteric fracture’  #9 #1 OR #2 OR #3 OR #4 OR #5 OR #6 OR #7 OR #8  #10 ’mortality’/exp OR ’mortality’  #11 ’fatality’/exp OR ’fatality’  #12 ’China’/exp OR ’China’  #13 ’mainland China’  #14 #10 OR #11  #15 #12 OR #13  #16 #9 AND #14 AND #15  #17 ’one year’  #18 ’twelve months’  #19 ’365 days’  #20 #17 OR #18 OR #19  #21 #16 AND #20  Access Date: from 01/12/2018 to 31/12/2018  Published time: from 01/01/2000 to 30/11/2018  **The Cochrane Library**  Search strategy  #1 Mesh descriptor:[hip fracture] this term only  #2 “Trochanteric Fractures” or “Fractures,Trochanteric” or “Intertrochanteric Fractures” or “Fractures, Intertrochanteric” or “Fractures, Hip” or “Fractures, Subtrochanteric” or “Subtrochanteric Fractures”  #3 #1 or #2  #4 Mesh descriptor:[mortality] this term only  #5 “Fatality” or “Death”  #6 #4 or #5  #7 Mesh descriptor:[China] this term only  #8 “People's Republic of China” or “mainland China”  #9 #7 or #8  #10 #3 and #6 and #9  Access Date: from 01/12/2018 to 31/12/2018  Published time: from 01/01/2000 to 30/11/2018 |
| --- |

**Table S1.** Search strategy in the six databases to identify studies reporting one-year mortality rates after hip fracture in mainland China.
