# Supplementary material for: Age-specific 1-year mortality rates after hip fracture based on the populations in mainland China between the years 2000 and 2018: a systematic analysis
Source: Arch Osteoporos. 2019 May 25;14(1):55. doi: 10.1007/s11657-019-0604-3 (PMC6535151; doi:10.1007/s11657-019-0604-3)
Supplement: Supplementary file 2 — (DOCX 14 kb) [file 11657_2019_604_MOESM2_ESM.docx]

| **Moderator** | **Hip fracture** | **Femoral intertrochanteric fracture** | **Femoral neck fracture** |
| --- | --- | --- | --- |
| **Age** | 1.062 (1.058~1.066)^*^ | 1.094 (1.071~1.117)^*^ | 1.108 (1.101~1.114)^*^ |
| **Setting:** |  |  |  |
| urban | Reference | Reference | Reference |
| mixed | 1.246 (1.067~1.456) | 0.732 (0.146~3.666) | 1.277 (1.124~1.451) |
| rural | 2.283 (0.509~10.247) | 1.714 (0.142~20.635) | ^#^ |
| **Region:** |  |  |  |
| North | Reference | Reference | Reference |
| Northeast | 2.759 (1.282~5.926) | 2.414 (1.980~2.942) |  |
| East | 0.641 (0.604~1.472) | 0.519 (0.509~0.529) | 0.949 (0.767~1.174) |
| South Central | 1.143 (0.782~1.671) | 1.108 (0.364~3.370) |  |
| Southwest | 1.551 (0.744~3.232) | 1.094 (0.030~39.925) | 1.996 (1.640~2.428) |
| Northwest | 0.458 (0.108~1.943) | 0.375 (0.041~3.449) | 0.586 (0.271~1.269) |
| **Study type:** |  |  |  |
| retrospective | Reference | Reference | Reference |
| prospective | 0.910 (0.456~1.816) | 0.914 (0.235~3.564) | 1.534 (0.273~8.637) |
| **Survey year** | 1.006 (1.005~1.008) | 0.927 (0.848~1.012) | 1.091 (1.073~1.110) |

| *: statistically significant  #: data were not available |
| --- |

**Table S2.** Odds ratios for hip fracture, femoral intertrochanteric fracture and femoral neck fracture in terms of age, setting, region, study type and survey year from multilevel univariable meta-regression models, with 95% confidence intervals.
