# Supplementary material for: Age-specific 1-year mortality rates after hip fracture based on the populations in mainland China between the years 2000 and 2018: a systematic analysis
Source: Arch Osteoporos. 2019 May 25;14(1):55. doi: 10.1007/s11657-019-0604-3 (PMC6535151; doi:10.1007/s11657-019-0604-3)
Supplement: Supplementary file 3 — (DOCX 27 kb) [file 11657_2019_604_MOESM3_ESM.docx]

| Study ID |  |
| --- | --- |
| H 01 | Yan H, Huang B, Zhong YL, et al. Effect of preoperative antiplatelet drugs on complications and fatality after femoral head replacement[J]. Shanxi Medical Journal 2016,45(16):1919-1922. |
| H 02 | Li RR. Analysis of 430 fragile hip fracture cases after 3 years follow-up[J]. Kunming Medical University 2017. |
| H 03 | Xie YZ. Analysis of the correlation between admission haemoglobin levels and postoperative recent mortality in elderly patients with hip fracture. Guangzhou University of Chinese Medicine 2017. |
| H 04 | Meng DF. Risk factors associated with the prognosis following hip fracture in elderly patients after 1 year. Hebei Medical University 2017. |
| H 05 | Xiong JB, Peng WQ, Sun HZ, et al. Retrospective analysis of relevant factors of death in postoperative elderly patients with hip fracture. Orthopedic Journal of China 2014,22(18):1666-1669. |
| H 06 | Hou ZH. The postoperative survival analysis of elderly patients with hip fracture and study of curative effect of different internal fixation among elderly patients with unstable intertrochanteric fracture. Zhejiang University 2014. |
| H 07 | Shen Y, Shen HL, Fang XT. Analysis on risk factors for mortality in elderly patients with femoral neck fractures underwent hemiarthroplasty[J]. Journal of Jilin University (Medicine Edition) 2013,39(03):574-577. |
| H 08 | Yu HW. Clinical study on the effect of surgical timing on the prognosis of elderly femoral intertrochanteric fracture[J]. China and Foreign Medical Treatment 2016,35(20):79-80. |
| H 09 | Cao C. The investigation of death causes after tuberosity fracture in senile patients and relative risk factors. Shandong University 2015. |
| H 10 | Li HX. Analysis of risk factors affecting mortality in elderly patients with femoral intertrochanteric fracture. Guangzhou University of Chinese Medicine 2014. |
| H 11 | Xu LZ. Relation between post-operative mortality and the time to femoral intertrochanteric fracture surgery in elderly patients[J]. Orthopedic Journal of China 2010,18(12):1045-1047. |
| H 12 | Yang SB.Analysis of related factors of death after arthroplasty in elderly patients with femoral neck fracture[J].Laboratory Medicine and Clinic 2016,13(9):1263-1265. |
| H 13 | Sun GF, Li XD, Yu HJ. Study on the influencing factors of the clinical outcome of patients with femoral neck fracture[J]. Chinese Journal of Primary Medicine and Pharmacy 2014,(16):2461-2462,2463. |
| H 14 | Zhang XY, An S, Huang J, et al. Risk factors for 1-year mortality in elderly patients after hip fracture surgery[J]. Chinese General Practice 2018,21(10):1198-1202. |
| H 15 | Xu C. The application of hemi hip arthroplasty for elderly femoral neck fracture and related research. Shanghai Jiao Tong University. 2015. |
| H 16 | Yao YF, Xue CX, Lv H, et al. Perioperative management of concomitant diseases and complications of hip fracture in patients aged 80 year and over[J]. [Chinese Journal of Geriatrics](http://g.wanfangdata.com.cn/details/javascript:void(0)) 2016,35(4):391-395. |
| H 17 | Yao Q, Yang J, Liu T, et al. Clinical characteristics of the 515 elderly with hip fracture in Ningbo[J].  Chinese Journal of Osteoporosis and Bone Mineral Research 2014,(2):144-148. |
| H 18 | Tang C, Fu SP. Effect of surgical timing of femoral intertrochanteric fractures on 1-year postoperative mortality in elderly[J]. China Journal of Orthopaedics and Traumatology 2017,30(7):602-606. |
| H 19 | Jiang HL, Guo KJ, Wang X, et al. Cemented versus uncemented hemiarthroplasty for unstable intertrochanteric fractures in the elderly[J]. [Chinese Journal of Tissue Engineering Research](http://g.wanfangdata.com.cn/details/javascript:void(0)) 2017,21(23):3622-3627. |
| H 20 | Zeng RX, Zhang YQ, Liu XF, et al. Observation on postoperative outcome of femoral intertrochanteric fracture in elderly patients with different operative time[J].Chinese Community Doctors 2011,13(17):87-88. |
| H 21 | Zhang Y, Qin XD, Xu YQ, et al. Risk factors related to mortality at one year after surgery for intertrochanteric fracture in the elderly[J]. Chinese Journal of Geriatric Orthopaedics and Rehabilitation (Electronic Edition) 2018,4(5):277-281 |
| H 22 | Cheng J, Feng SM, Ma Y, et al. Timing of surgery for elderly peritrochanteric fractures and in-hospital mortality[J]. Orthopedic Journal of China 2016,24(8):673-676. |
| H 23 | Sun Q, Tong PJ, Xu BB, et al. Risk factors for 1-year mortality in elderly patients after intertrochanteric fracture surgery: a retrospective study of 1854 cases[J]. Chinese Journal of Orthopedic Trauma 2013,15(5):371-376. |
| H 24 | Sun CS, Zheng X, Guo KJ, et al. Relationship between low basal metabolic rate and mortality in older adults with hip fractures[J]. Chinese Journal of Tissue Engineering Research 2018,22(16):2467-2471. |
| H 25 | Li CY, Zhang XC, Zhen X, et al. Risk factors for 1-year mortality in elderly patients after hip fracture surgery[J]. [Journal of Practical Orthopaedics](http://g.wanfangdata.com.cn/details/javascript:void(0)) 2017,23(9):802-806. |
| H 26 | Zhu YJ. Analysis of nutritional status and prognosis in elderly patients with hip fracture. Medical College of Southeast University 2016. |
| H 27 | Cao LH, Chen X, Weng XD, et al. Surgical treatment options for hip fractures in elderly patients aged 80 years old and over: a report of 915 cases[J]. Academic Journal of Second Military Medical University 2017,38(4):409-414. |
| H 28 | Lu WL, Dewan SK, Lin WL, et al. Assessment on peri-operative risk and 1-year outcomes in elderly patients with hip fracture[J]. Chinese Journal of Multiple Organ Diseases in the Elderly 2014,(6):440-444. |
| H 29 | Yang LH, An LK, Liu W, et al. Perioperative management and risk factors affecting the prognosis of hip fracture surgery in elderly patients[J]. Chinese Journal of Geriatrics 2014,33(5):507-509. |
| H 30 | Li J, Long AH, Zhang LC, et al. Effect of pre-operative serum albumin level and total lymphocyte count on outcomes of elderly patients with hip fracture[J]. Chinese Journal of Multiple Organ Diseases in the Elderly 2014,(4):255-258. |
| H 31 | Mao D. Therapeutic effects for the treatment of senile hip fractures[J]. China Journal of Orthopaedics and Traumatology 2009,22(7):511-512. |
| H 32 | Wang ZZ. Mortality analysis following intertrochanteric fracture surgery in patients. Xin Jiang Medical University. 2018 |
| H 33 | Liu XF. The Predictive Value of Full Blood Count in Short-term Mortality of Brittle Hip Fracture. Fujian Medical University. 2018 |
| H 34 | Li Z, Liu Y, Huang FG. Risk factors of death in elderly patients with femoral neck fracture over 90 years of age[J]. Chinese Journal of Reparative and Reconstructive Surgery 2018,32(8):1102-1104. |
| H35 | Tan ZW, Qiao J, Zhang J. Comparison of three different surgical methods in the treatment of displaced femoral neck fractures in elderly patients[J]. Chinese Journal of the Frontiers of Medical Science (Electronic Version) 2017,9(8):51-54. |
| H 36 | Meng HL, Wang KZ, Wang CS, et al. Cemented versus uncemented bipolar hemiarthroplasty for femoral neck fractures in the elderly[J]. Journal of Clinical Rehabilitative Tissue Engineering Research 2009,13(22):4231-4236. |
| H 37 | Wang LQ, Li Y, Liu CG, et al. Risk factors for mortality in nonagenarians with femoral neck fractures undergoing joint replacement[J]. National Medical Journal of China 2015,95(11):832-835. |
| H 38 | Li TZ, Zhang BK, Mo XL, et al. The comparative analysis of extramedullary to intramedullary fixations for unstable femur intertrochanteric fractures in elderly patients[J]. Chinese Journal of Geriatric Orthopaedics and Rehabilitation (Electronic Edition) 2017,3(1):17-21. |
| H 39 | Chen ZB, Wang JK, Guang WY. Analysis of the intermediate-term survival after hip fracture surgery in senile patients[J]. The Medical Forum 2015,(30):4177-4179. |
| H 40 | Zhang SL, Zheng LB, Hou ZH, et al. The postoperative survival rate analysis of elderly patients with hip fracture[J]. Chinese Journal of Geriatrics 2015,34(7):778-781. |
| H 41 | Feng ML, Shen HL. Predictors of one year mortality in the elderly patients with hip fracture following poststroke hemiplegia[J]. Chinese Journal of Orthopedic Trauma 2012,14(2):93-97. |
| H 42 | Wang Y, Wang M, Chen H, et al. Early out-of-bed functional exercise benefits elderly patients following hip fracture: a retrospective cohort study[J]. [Tohoku J Exp Med](https://www.ncbi.nlm.nih.gov/pubmed/?term=Early+Out-of-Bed+Functional+Exercise+Benefits+Elderly+Patients+Following%C2%A0Hip+Fracture:+A+Retrospective+Cohort+Study) 2018;246(4):205-212. |
| H 43 | Li SG, Sun T, Liu Z. Excess mortality of 1 year in elderly hip fracture patients compared with the general population in Beijing, China[J]. Arch Osteoporos 2016,11(1): 35. |
| H 44 | Lu J, Chen YY, Zhang L, et al. Laboratory nutritional parameters predict one-year mortality in elderly patients with intertrochanteric fracture[J]. Asia Pac J Clin Nutr 2016,25(3):457-463. |
| H 45 | Zhao P, Lian X, Dou X, et al. Intertrochanteric hip fracture surgery in Chinese: risk factors for predicting mortality[J]. Int J Clin Exp Med 2015,8(2):2789-2793. |
| H 46 | Liu Y, Peng M, Lin L, et al. Relationship between American Society of Anesthesiologists (ASA) grade and 1-year mortality in nonagenarians undergoing hip fracture surgery[J].Osteoporosis International 2015,26(3):1029-1033. |
| H 47 | Li SG, Sun TS, Liu Z, et al. Factors influencing postoperative mortality one year after surgery for hip fracture in Chinese elderly population[J]. Chinese Medical Journal(English) 2013,126(14):2715-2719. |
| H 48 | Shi L, Wang XC, Wang YS. Artificial neural network models for predicting 1-year mortality in elderly patients with intertrochanteric fractures in China First Affiliated Hospital, Liaoning Medical University[J]. Brazilian Journal of Medical and Biological Research 2013,46(11):993-999. |
| H 49 | Wang XW, Sun TS, Liu Z, et al. Risk factors related to prognosis following hip fracture surgery  in the aged patients[J]. [Chinese Journal of Orthopaedic Trauma](http://g.wanfangdata.com.cn/details/javascript:void(0)) 2011,13(9):811-816. |
| H 50 | Wu W, Huang WJ, Shen B, et al. Prospective study of the operative treatment of intertrochanteric fracture in elderly persons over 80 years[J]. Orthopedic Journal of China 2010,18(17):1481-1483 |
| H 51 | Wang XF, Zhao YJ, Wang XY, et al. Cemented bipolar femoral component arthroplasty compared with intramedullary fixation for treatment of unstable femoral intertrochanteric fractures[J]. Chinese Journal of Bone and Joint Injury 2008,23(3):189-191. |
| H 52 | Wu B, Lin MX, Shen NJ, et al. Clinical outcome of hip hemiarthroplasty on intertrochanteric  fracture in elderly patients[J]. Journal of Practice Orthopaedics 2018,24(12):1073-1076,1092. |
| H 53 | Wang YY, Wang MS, Huang WB, et al. Analysis of the opportunity and effect of functional exercise after the hip arthroplasty in the elderly patients[J]. Guangdong Medical Journal 2016,37 (12):3236-3239. |
| H 54 | Dai B, Luo HT, Meng XD, et al. Influnence of hip fracture type on outcome in elderly hip  fracture[J]. Chinese Journal of Clinicians (Electronic Version) 2010,4(5):630-634. |

**Table S3.** Full list of the 54 included studies on one-year mortality rates after hip fracture in mainland China.
