# Supplementary material for: Age-specific 1-year mortality rates after hip fracture based on the populations in mainland China between the years 2000 and 2018: a systematic analysis
Source: Arch Osteoporos. 2019 May 25;14(1):55. doi: 10.1007/s11657-019-0604-3 (PMC6535151; doi:10.1007/s11657-019-0604-3)
Supplement: Supplementary file 4 — (DOCX 29 kb) [file 11657_2019_604_MOESM4_ESM.docx]

| **Author** | **Publication Year** | **Study Period** | **Province** | **Geographic Regions** | **Setting** | **Sample Size（n）** | **Mean Age (Y)** | **Study Types** | **One-year Mortality Rate** |
| --- | --- | --- | --- | --- | --- | --- | --- | --- | --- |
| Yan H | 2016 | 2011-2013 | Chongqing Municipality | Southwest | Mixed | 133 | 82.8 | retrospective | 0.203 |
| Li RR | 2017 | 2013-2013 | Yunnan | Southwest | Urban | 430 | 75 | retrospective | 0.253 |
| Xie YZ | 2017 | 2013-2015 | Guangdong | South Central | Urban | 308 | 80.2 | retrospective | 0.127 |
| Meng DF | 2017 | 2015-2016 | Hebei | North | Urban | 300 | 80 | retrospective | 0.153 |
| Xiong JB | 2014 | 2008-2011 | Guangxi | South Central | Mixed | 196 | 78.5 | retrospective | 0.179 |
| Hou ZH | 2014 | 2009-2011 | Zhejiang | East | Urban | 255 | 79.1 | retrospective | 0.102 |
| Shen Y | 2013 | 2007-2011 | Beijing Municipality | North | Urban | 193 | 74.1 | retrospective | 0.067 |
| Yu HW | 2016 | 2015-2016 | Sichuan | Southwest | Mixed | 68 | 78.9 | retrospective | 0.176 |
| Cao C | 2015 | 2009-2014 | Shandong | East | Urban | 132 | 82.5 | retrospective | 0.265 |
| Li HX | 2014 | 2012-2013 | Guangdong | South Central | Urban | 152 | 80.5 | retrospective | 0.224 |
| Xu LS | 2010 | 2005-2008 | Beijing Municipality | North | Urban | 172 | 78.6 | retrospective | 0.203 |
| Yang SB | 2016 | 2011-2014 | Yunnan | Southwest | Mixed | 90 | 74.5 | retrospective | 0.100 |
| Sun GF | 2014 | 2009-2012 | Shandong | East | Mixed | 180 | 75.3 | retrospective | 0.067 |
| Zhang XY | 2018 | 2014-2015 | Beijing Municipality | North | Urban | 234 | 74.5 | retrospective | 0.115 |
| Xu C | 2015 | 2006-2012 | Shanghai Municipality | East | Urban | 258 | 85 | retrospective | 0.093 |
| Yao YF | 2016 | 2008-2013 | Anhui | East | Urban | 73 | 84.5 | retrospective | 0.219 |
| Yao Q | 2014 | 2010-2012 | Zhejiang | East | Mixed | 497 | 77.1 | retrospective | 0.054 |
| Tang C | 2017 | 2012-2014 | Shanghai Municipality | East | Urban | 350 | 79.5 | retrospective | 0.071 |
| Jiang HL | 2017 | 2009-2014 | Shandong | East | Mixed | 93 | 80.4 | retrospective | 0.075 |
| Zeng RX | 2011 | 2008-2011 | Guangdong | South Central | Rural | 129 | 84.3 | retrospective | 0.248 |
| Zhang Y | 2018 | 2015-2017 | Jiangsu | East | Urban | 161 | 75 | retrospective | 0.124 |
| Cheng J | 2016 | 2013-2014 | Jiangsu | East | Mixed | 98 | 71.9 | retrospective | 0.163 |
| Sun Q | 2013 | 2005-2011 | Zhejiang | East | Urban | 1854 | 79.4 | retrospective | 0.160 |
| Sun CS | 2018 | 2014-2016 | Jiangsu | East | Mixed | 507 | 77.3 | retrospective | 0.134 |
| Li CY | 2017 | 2013-2014 | Jiangsu | East | Mixed | 819 | 76.3 | retrospective | 0.170 |
| Zhu YJ | 2016 | 2013-2014 | Jiangsu | East | Urban | 120 | 72.5 | retrospective | 0.100 |
| Cao LH | 2017 | 2007-2016 | Shanghai Municipality | East | Urban | 915 | 86.6 | retrospective | 0.015 |
| Lu WL | 2014 | 2010-2010 | Shanghai Municipality | East | Urban | 234 | 80.1 | retrospective | 0.137 |
| Yang LH | 2014 | 2009-2013 | Beijing Municipality | North | Mixed | 112 | 76.2 | retrospective | 0.214 |
| Li J | 2014 | 2006-2010 | Beijing Municipality | North | Urban | 640 | 72.5 | retrospective | 0.100 |
| Mao D | 2009 | 2001-2006 | Jiangsu | East | Rural | 136 | 78.9 | retrospective | 0.235 |
| Wang ZZ | 2018 | 2012-2016 | Xinjiang Uyghur Autonomous Region | Northwest | Urban | 156 | 77.4 | retrospective | 0.071 |
| Liu XF | 2016 | 2010-2012 | Fujian | East | Mixed | 231 | 76.9 | retrospective | 0.160 |
| Li Z | 2018 | 2011-2016 | Sichuan | Southwest | Urban | 64 | 92.3 | retrospective | 0.234 |
| Tan ZW | 2017 | 2013-2015 | Sichuan | Southwest | Mixed | 130 | 73.1 | retrospective | 0.069 |
| Meng HL | 2009 | 2004-2005 | Shananxi | Northwest | Urban | 131 | 79 | retrospective | 0.053 |
| Wang LQ | 2015 | 2007-2011 | Beijing Municipality | North | Urban | 232 | 72.4 | retrospective | 0.060 |
| Li TZ | 2017 | 2012-2013 | Shanghai Municipality | East | Urban | 60 | 92 | retrospective | 0.217 |
| Chen ZB | 2015 | 2010-2013 | Jiangsu | East | Mixed | 55 | 81.8 | retrospective | 0.109 |
| Zhang SL | 2015 | 2008-2011 | Zhejiang | East | Urban | 255 | 79.1 | retrospective | 0.102 |
| Feng ML | 2012 | 2000-2007 | Beijing Municipality | North | Urban | 1379 | 76.4 | retrospective | 0.118 |
| Wang Y | 2018 | 2007-2017 | Guangdong | South Central | Mixed | 1022 | 83 | retrospective | 0.155 |
| Li SG | 2016 | 2013-2013 | Beijing Municipality | North | Mixed | 4504 | 77.3 | retrospective | 0.234 |
| Lu J | 2016 | 2009-2010 | Jiangsu | East | Urban | 149 | 80.9 | retrospective | 0.309 |
| Zhao P | 2015 | 2005-2010 | Zhejiang | East | Urban | 491 | 70 | retrospective | 0.177 |
| Liu Y | 2015 | 2000-2012 | Hebei | North | Urban | 327 | 93.7 | retrospective | 0.116 |
| Li SG | 2013 | 2009-2011 | Beijing Municipality | North | Urban | 153 | 75.6 | retrospective | 0.124 |
| Shi L | 2013 | 2001-2010 | Liaoning | Northeast | Urban | 2150 | 81.6 | retrospective | 0.271 |
| Wang XW | 2017 | 2006-2008 | Beijing Municipality | North | Urban | 127 | 83.5 | retrospective | 0.339 |
| Wu W | 2010 | 2006-2007 | Shanghai Municipality | East | Urban | 51 | 84.5 | prospective | 0.059 |
| Wang XF | 2008 | 2002-2005 | Beijing Municipality | North | Urban | 68 | 78 | prospective | 0.029 |
| Wu B | 2018 | 2014-2017 | Hainan | South Central | Urban | 163 | 78.2 | prospective | 0.307 |
| Wang YY | 2016 | 2005-2015 | Guangdong | South Central | Urban | 1022 | 83.2 | prospective | 0.096 |
| Dai B | 2007 | 2007-2008 | Beijing Municipality | North | Urban | 41 | 79.8 | prospective | 0.122 |

**Table S4.** The detailed characteristics of 54 studies included in this systematic review.
