# Supplementary material for: Age-specific 1-year mortality rates after hip fracture based on the populations in mainland China between the years 2000 and 2018: a systematic analysis
Source: Arch Osteoporos. 2019 May 25;14(1):55. doi: 10.1007/s11657-019-0604-3 (PMC6535151; doi:10.1007/s11657-019-0604-3)
Supplement: Supplementary file 5 — (DOCX 24 kb) [file 11657_2019_604_MOESM5_ESM.docx]

| **Study ID** | | **Selection** | | | | **Comparability** | | **Outcome** | | | | **NOS Scores** | |
| --- | --- | --- | --- | --- | --- | --- | --- | --- | --- | --- | --- | --- | --- |
|  |  | Representativeness of the exposed cohort | Selection of the non exposed cohort | Ascertainment of exposure | Demonstration that outcome of interest was not present at start of study | | Comparability of cohorts on the basis of the design or analysis | | Assessment of outcome | Was follow-up long enough for outcomes to occur | Adequacy of follow up of cohorts |  |  |
| H 01 | 1 | 1 | 1 | 1 | | 0 | | 0 | 0 | 0 | | 4 | |
| H 02 | 1 | 1 | 1 | 1 | | 0 | | 0 | 0 | 0 | | 4 | |
| H 03 | 1 | 1 | 1 | 1 | | 0 | | 1 | 1 | 0 | | 6 | |
| H 04 | 1 | 1 | 1 | 1 | | 0 | | 1 | 1 | 1 | | 7 | |
| H 05 | 1 | 1 | 0 | 1 | | 0 | | 0 | 1 | 1 | | 5 | |
| H 06 | 1 | 1 | 1 | 1 | | 0 | | 1 | 1 | 1 | | 7 | |
| H 07 | 1 | 1 | 1 | 1 | | 0 | | 0 | 0 | 1 | | 5 | |
| H 08 | 1 | 1 | 1 | 1 | | 0 | | 0 | 0 | 0 | | 4 | |
| H 09 | 1 | 0 | 0 | 1 | | 1 | | 0 | 1 | 1 | | 5 | |
| H 10 | 1 | 0 | 0 | 1 | | 1 | | 0 | 1 | 1 | | 5 | |
| H 11 | 1 | 1 | 1 | 1 | | 0 | | 0 | 0 | 0 | | 4 | |
| H 12 | 1 | 1 | 1 | 1 | | 0 | | 0 | 0 | 1 | | 5 | |
| H 13 | 1 | 1 | 1 | 1 | | 0 | | 0 | 0 | 0 | | 4 | |
| H 14 | 1 | 0 | 0 | 1 | | 1 | | 0 | 1 | 1 | | 5 | |
| H 15 | 1 | 0 | 0 | 1 | | 1 | | 0 | 1 | 1 | | 5 | |
| H 16 | 1 | 0 | 0 | 1 | | 0 | | 0 | 1 | 1 | | 4 | |
| H 17 | 1 | 0 | 0 | 1 | | 0 | | 1 | 0 | 1 | | 4 | |
| H 18 | 1 | 1 | 1 | 1 | | 1 | | 0 | 0 | 0 | | 5 | |
| H 19 | 1 | 1 | 1 | 1 | | 0 | | 1 | 1 | 1 | | 7 | |
| H 20 | 1 | 1 | 1 | 1 | | 0 | | 0 | 0 | 0 | | 4 | |
| H 21 | 1 | 0 | 0 | 1 | | 1 | | 0 | 1 | 0 | | 4 | |
| H 22 | 1 | 1 | 1 | 1 | | 0 | | 0 | 0 | 0 | | 4 | |
| H 23 | 1 | 0 | 0 | 1 | | 1 | | 0 | 1 | 1 | | 5 | |
| H 24 | 1 | 0 | 0 | 1 | | 0 | | 0 | 1 | 1 | | 4 | |
| H 25 | 1 | 0 | 0 | 1 | | 1 | | 0 | 1 | 1 | | 5 | |
| H 26 | 1 | 0 | 0 | 1 | | 1 | | 0 | 1 | 1 | | 5 | |
| H 27 | 1 | 1 | 1 | 1 | | 0 | | 0 | 1 | 0 | | 5 | |
| H 28 | 1 | 0 | 0 | 1 | | 1 | | 0 | 1 | 1 | | 5 | |
| H 29 | 1 | 1 | 1 | 1 | | 0 | | 0 | 1 | 0 | | 5 | |
| H 30 | 1 | 0 | 0 | 1 | | 1 | | 0 | 1 | 1 | | 5 | |
| H 31 | 1 | 1 | 1 | 1 | | 0 | | 0 | 1 | 0 | | 5 | |
| H 32 | 1 | 0 | 0 | 1 | | 1 | | 0 | 1 | 0 | | 4 | |
| H 33 | 1 | 1 | 1 | 1 | | 1 | | 0 | 1 | 1 | | 7 | |
| H 34 | 1 | 0 | 0 | 1 | | 1 | | 0 | 0 | 1 | | 4 | |
| H35 | 1 | 1 | 1 | 1 | | 1 | | 0 | 1 | 0 | | 6 | |
| H 36 | 1 | 1 | 1 | 1 | | 0 | | 0 | 0 | 0 | | 4 | |
| H 37 | 1 | 1 | 1 | 1 | | 0 | | 0 | 1 | 1 | | 6 | |
| H 38 | 1 | 1 | 1 | 1 | | 0 | | 0 | 0 | 0 | | 4 | |
| H 39 | 1 | 0 | 0 | 1 | | 1 | | 0 | 1 | 0 | | 4 | |
| H 40 | 1 | 0 | 0 | 1 | | 1 | | 0 | 1 | 1 | | 5 | |
| H 41 | 1 | 1 | 1 | 1 | | 1 | | 0 | 0 | 0 | | 5 | |
| H 42 | 1 | 1 | 1 | 1 | | 1 | | 0 | 1 | 1 | | 7 | |
| H 43 | 1 | 1 | 1 | 1 | | 1 | | 0 | 0 | 1 | | 6 | |
| H 44 | 1 | 1 | 1 | 1 | | 1 | | 0 | 1 | 1 | | 7 | |
| H 45 | 1 | 0 | 0 | 1 | | 1 | | 0 | 1 | 0 | | 4 | |
| H 46 | 1 | 0 | 0 | 1 | | 1 | | 0 | 1 | 0 | | 4 | |
| H 47 | 1 | 0 | 0 | 1 | | 1 | | 0 | 1 | 1 | | 5 | |
| H 48 | 1 | 0 | 0 | 1 | | 1 | | 0 | 1 | 0 | | 4 | |
| H 49 | 1 | 0 | 0 | 1 | | 1 | | 0 | 1 | 1 | | 5 | |
| H 50 | 1 | 1 | 1 | 1 | | 1 | | 0 | 0 | 1 | | 6 | |
| H 51 | 1 | 1 | 1 | 1 | | 1 | | 0 | 0 | 1 | | 6 | |
| H 52 | 1 | 1 | 1 | 1 | | 1 | | 0 | 1 | 1 | | 7 | |
| H 53 | 1 | 1 | 1 | 1 | | 1 | | 0 | 1 | 1 | | 7 | |
| H 54 | 1 | 1 | 1 | 1 | | 0 | | 0 | 1 | 1 | | 6 | |

**Table S5.** The Newcastle-Ottawa Scale (NOS) of 54 studies included in this systematic analysis.
