# Supplementary material for: Age-specific 1-year mortality rates after hip fracture based on the populations in mainland China between the years 2000 and 2018: a systematic analysis
Source: Arch Osteoporos. 2019 May 25;14(1):55. doi: 10.1007/s11657-019-0604-3 (PMC6535151; doi:10.1007/s11657-019-0604-3)
Supplement: Supplementary file 6 — (DOCX 21 kb) [file 11657_2019_604_MOESM6_ESM.docx]

|  | Study | Proportion | 95%CI | | tau^2 | I^2 |
| --- | --- | --- | --- | --- | --- | --- |
| Omitting | Yan H 2016 | 0.1385 | 0.1214 | 0.1577 | 0.2620 | 93.2% |
| Omitting | Li RR 2017 | 0.1376 | 0.1205 | 0.1567 | 0.2631 | 93.0% |
| Omitting | Xie YZ 2017 | 0.1399 | 0.1226 | 0.1591 | 0.2610 | 93.1% |
| Omitting | Meng DF 2017 | 0.1393 | 0.1220 | 0.1586 | 0.2633 | 93.1% |
| Omitting | Xiong JB 2014 | 0.1389 | 0.1217 | 0.1581 | 0.2628 | 93.2% |
| Omitting | Hou ZH 2014 | 0.1405 | 0.1232 | 0.1597 | 0.2582 | 93.1% |
| Omitting | Shen Y 2013 | 0.1414 | 0.1241 | 0.1606 | 0.2553 | 93.0% |
| Omitting | Yu HW 2016 | 0.1391 | 0.1219 | 0.1582 | 0.2608 | 93.2% |
| Omitting | Cao C 2015 | 0.1377 | 0.1207 | 0.1567 | 0.2604 | 93.1% |
| Omitting | Li HX 2014 | 0.1382 | 0.1211 | 0.1573 | 0.2620 | 93.1% |
| Omitting | Xu LS 2010 | 0.1385 | 0.1213 | 0.1576 | 0.2626 | 93.1% |
| Omitting | Yang SB 2016 | 0.1403 | 0.1231 | 0.1595 | 0.2592 | 93.1% |
| Omitting | Sun GF 2014 | 0.1414 | 0.1241 | 0.1606 | 0.2555 | 93.0% |
| Omitting | Zhang XY 2018 | 0.1401 | 0.1229 | 0.1594 | 0.2597 | 93.1% |
| Omitting | Xu C 2015 | 0.1407 | 0.1235 | 0.1600 | 0.2571 | 93.0% |
| Omitting | Yao YF 2016 | 0.1385 | 0.1214 | 0.1575 | 0.2609 | 93.1% |
| Omitting | Yao Q 2014 | 0.1424 | 0.1253 | 0.1614 | 0.2447 | 92.7% |
| Omitting | Tang C 2017 | 0.1415 | 0.1243 | 0.1607 | 0.2523 | 92.9% |
| Omitting | Jiang HL 2017 | 0.1408 | 0.1236 | 0.1600 | 0.2580 | 93.1% |
| Omitting | Zeng RX 2011 | 0.1379 | 0.1209 | 0.1570 | 0.2610 | 93.1% |
| Omitting | Zhang Y 2018 | 0.1399 | 0.1227 | 0.1591 | 0.2603 | 93.1% |
| Omitting | Cheng J 2016 | 0.1392 | 0.1220 | 0.1584 | 0.2611 | 93.2% |
| Omitting | Sun Q 2013 | 0.1388 | 0.1211 | 0.1587 | 0.2838 | 93.1% |
| Omitting | Sun CS 2018 | 0.1397 | 0.1224 | 0.1590 | 0.2630 | 93.1% |
| Omitting | Li CY 2017 | 0.1388 | 0.1214 | 0.1583 | 0.2718 | 93.1% |
| Omitting | Zhu YJ 2016 | 0.1404 | 0.1232 | 0.1596 | 0.2590 | 93.1% |
| Omitting | Cao LH 2017 | 0.1453 | 0.1285 | 0.1639 | 0.2248 | 92.1% |
| Omitting | Lu WL 2014 | 0.1397 | 0.1224 | 0.1589 | 0.2615 | 93.1% |
| Omitting | LH 2014 | 0.1384 | 0.1213 | 0.1575 | 0.2615 | 93.1% |
| Omitting | Li J 2014 | 0.1407 | 0.1235 | 0.1598 | 0.2550 | 92.9% |
| Omitting | Mao D 2009 | 0.1381 | 0.1210 | 0.1571 | 0.2615 | 93.1% |
| Omitting | Wang ZZ 2018 | 0.1412 | 0.1239 | 0.1604 | 0.2564 | 93.0% |
| Omitting | Liu XF 2018 | 0.1392 | 0.1219 | 0.1584 | 0.2628 | 93.1% |
| Omitting | Li Z 2018 | 0.1383 | 0.1212 | 0.1573 | 0.2606 | 93.1% |
| Omitting | Tan ZW 2017 | 0.1411 | 0.1239 | 0.1603 | 0.2569 | 93.1% |
| Omitting | Meng HL 2009 | 0.1415 | 0.1243 | 0.1607 | 0.2558 | 93.0% |
| Omitting | Wang LQ 2015 | 0.1413 | 0.1240 | 0.1605 | 0.2556 | 93.0% |
| Omitting | Li TZ 2017 | 0.1385 | 0.1215 | 0.1576 | 0.2607 | 93.1% |
| Omitting | Chen ZB 2015 | 0.1401 | 0.1229 | 0.1592 | 0.2597 | 93.1% |
| Omitting | Zhang SL 2015 | 0.1405 | 0.1232 | 0.1597 | 0.2582 | 93.1% |
| Omitting | Feng ML 2012 | 0.1401 | 0.1229 | 0.1594 | 0.2596 | 92.8% |
| Omitting | Wang Y 2018 | 0.1391 | 0.1216 | 0.1587 | 0.2718 | 93.1% |
| Omitting | Li SG 2016 | 0.1375 | 0.1198 | 0.1574 | 0.2883 | 92.1% |
| Omitting | Lu J 2016 | 0.1372 | 0.1203 | 0.1560 | 0.2576 | 93.0% |
| Omitting | Zhao P 2015 | 0.1388 | 0.1214 | 0.1581 | 0.2674 | 93.2% |
| Omitting | Liu Y 2015 | 0.1401 | 0.1229 | 0.1594 | 0.2597 | 93.1% |
| Omitting | Li SG 2013 | 0.1399 | 0.1227 | 0.1591 | 0.2603 | 93.1% |
| Omitting | Shi L 2013 | 0.1375 | 0.1207 | 0.1562 | 0.2521 | 91.8% |
| Omitting | Wang XW 2017 | 0.1369 | 0.1201 | 0.1557 | 0.2556 | 93.0% |
| Omitting | Wu W 2010 | 0.1407 | 0.1235 | 0.1599 | 0.2584 | 93.1% |
| Omitting | Wang XF 2008 | 0.1412 | 0.1240 | 0.1603 | 0.2572 | 93.1% |
| Omitting | Wu B 2018 | 0.1372 | 0.1203 | 0.1560 | 0.2575 | 93.0% |
| Omitting | Wang YY 2016 | 0.1409 | 0.1238 | 0.1599 | 0.2497 | 92.7% |
| Omitting | Dai B 2007 | 0.1387 | 0.1226 | 0.1579 | 0.2613 | 93.2% |

**Table S6.** Leave-one-out sensitivity analysis of one-year mortality rates after hip fracture.
