# Supplementary material for: Age-specific 1-year mortality rates after hip fracture based on the populations in mainland China between the years 2000 and 2018: a systematic analysis
Source: Arch Osteoporos. 2019 May 25;14(1):55. doi: 10.1007/s11657-019-0604-3 (PMC6535151; doi:10.1007/s11657-019-0604-3)
Supplement: Supplementary file 7 — (DOCX 15 kb) [file 11657_2019_604_MOESM7_ESM.docx]

|  | Study | Proportion | 95%CI | | tau^2 | I^2 |
| --- | --- | --- | --- | --- | --- | --- |
| Omitting | Yu HW 2016 | 0.1745 | 0.1417 | 0.2130 | 0.2188 | 90.7% |
| Omitting | Cao C 2015 | 0.1697 | 0.1373 | 0.2079 | 0.2223 | 90.6% |
| Omitting | Li HX 2014 | 0.1715 | 0.1387 | 0.2103 | 0.2253 | 90.8% |
| Omitting | Xu LS 2010 | 0.1726 | 0.1395 | 0.2115 | 0.2258 | 90.8% |
| Omitting | Tang C 2017 | 0.1860 | 0.1540 | 0.2227 | 0.1737 | 88.4% |
| Omitting | Jiang HL 2017 | 0.1809 | 0.1480 | 0.2193 | 0.2060 | 90.3% |
| Omitting | Zeng RX 2011 | 0.1705 | 0.1379 | 0.2089 | 0.2233 | 90.7% |
| Omitting | Zhang Y 2018 | 0.1782 | 0.1451 | 0.2170 | 0.2123 | 90.4% |
| Omitting | Cheng J 2016 | 0.1752 | 0.1422 | 0.2139 | 0.2188 | 90.7% |
| Omitting | Sun Q 2013 | 0.1748 | 0.1404 | 0.2154 | 0.2410 | 88.1% |
| Omitting | Wang ZZ 2018 | 0.1830 | 0.1501 | 0.2211 | 0.1976 | 89.8% |
| Omitting | Li TZ 2017 | 0.1726 | 0.1401 | 0.2109 | 0.2196 | 90.8% |
| Omitting | Lu J 2016 | 0.1678 | 0.1360 | 0.2053 | 0.2163 | 90.3% |
| Omitting | Zhao P 2015 | 0.1737 | 0.1398 | 0.2138 | 0.2368 | 90.5% |
| Omitting | Shi L 2013 | 0.1694 | 0.1380 | 0.2061 | 0.2010 | 84.8% |
| Omitting | Wu W 2010 | 0.1797 | 0.1470 | 0.2178 | 0.2091 | 90.4% |
| Omitting | Wang XF 2008 | 0.1809 | 0.1484 | 0.2188 | 0.2046 | 90.3% |
| Omitting | Wu B 2018 | 0.1678 | 0.1360 | 0.2054 | 0.2167 | 90.3% |
| Omitting | Dai B 2007 | 0.1714 | 0.1391 | 0.2093 | 0.2189 | 90.7% |

**Table S7.** Leave-one-out sensitivity analysis of one-year mortality rates after femoral intertrochanteric fracture.
