# Supplementary material for: Age-specific 1-year mortality rates after hip fracture based on the populations in mainland China between the years 2000 and 2018: a systematic analysis
Source: Arch Osteoporos. 2019 May 25;14(1):55. doi: 10.1007/s11657-019-0604-3 (PMC6535151; doi:10.1007/s11657-019-0604-3)
Supplement: Supplementary file 8 — (DOCX 14 kb) [file 11657_2019_604_MOESM8_ESM.docx]

|  | Study | Proportion | 95%CI | | tau^2 | | I^2 |
| --- | --- | --- | --- | --- | --- | --- | --- |
| Omitting | Yan H 2016 | 0.0889 | 0.0653 | 0.1199 | | 0.1580 | 62.5% |
| Omitting | Shen Y 2013 | 0.1026 | 0.0708 | 0.1464 | | 0.2854 | 76.6% |
| Omitting | Yang SB 2016 | 0.0980 | 0.0669 | 0.1412 | | 0.3063 | 78.5% |
| Omitting | Sun GF 2014 | 0.1026 | 0.0709 | 0.1463 | | 0.2842 | 76.7% |
| Omitting | Xu C 2015 | 0.0987 | 0.0660 | 0.1451 | | 0.3434 | 78.3% |
| Omitting | Li Z 2018 | 0.0887 | 0.0641 | 0.1214 | | 0.1893 | 68.6% |
| Omitting | Tan ZW 2017 | 0.1018 | 0.0703 | 0.1454 | | 0.2882 | 77.5% |
| Omitting | Meng HL 2009 | 0.1041 | 0.0729 | 0.1465 | | 0.2637 | 76.2% |
| Omitting | Wang LQ 2015 | 0.1020 | 0.0700 | 0.1462 | | 0.2934 | 76.9% |
| Omitting | Dai B 2007 | 0.0965 | 0.0666 | 0.1379 | | 0.2935 | 78.5% |

**Table S8.**  Leave-one-out sensitivity analysis of one-year mortality rates after femoral neck fracture.
