# Supplementary figures and images for: Age-specific 1-year mortality rates after hip fracture based on the populations in mainland China between the years 2000 and 2018: a systematic analysis
Source: Arch Osteoporos. 2019 May 25;14(1):55. doi: 10.1007/s11657-019-0604-3 (PMC6535151; doi:10.1007/s11657-019-0604-3)

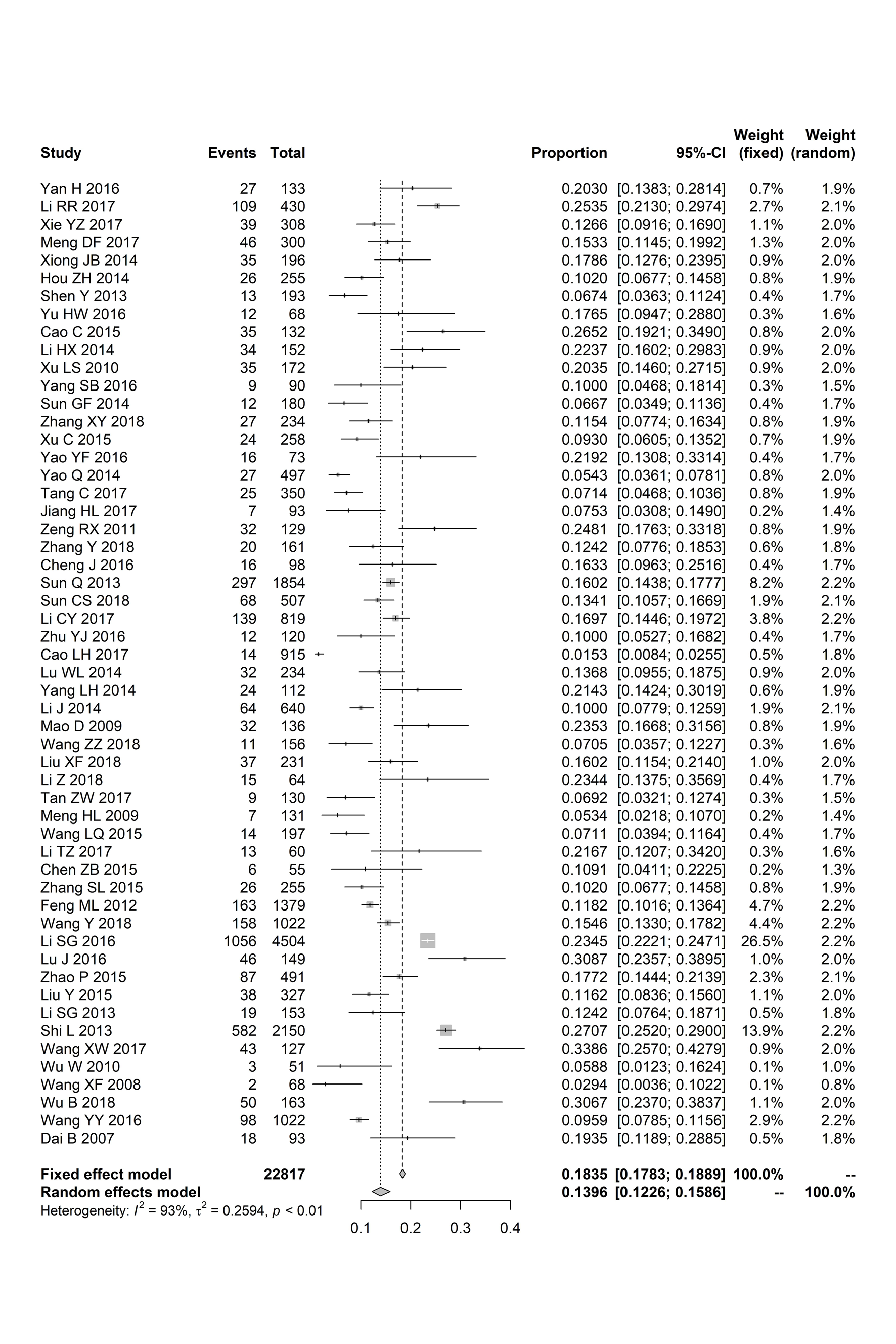

Supplement: Supplementary file 9 — Forest plot of the one-year mortality rate after hip fracture in mainland China. A total of 54 studies were included in the meta-analysis. The one-year mortality rate was calculated as 13.96% (95% CI 12.26% to 15.86%) using a random-effects model. (PNG 1697 kb) [file 11657_2019_604_Fig5_ESM.png]

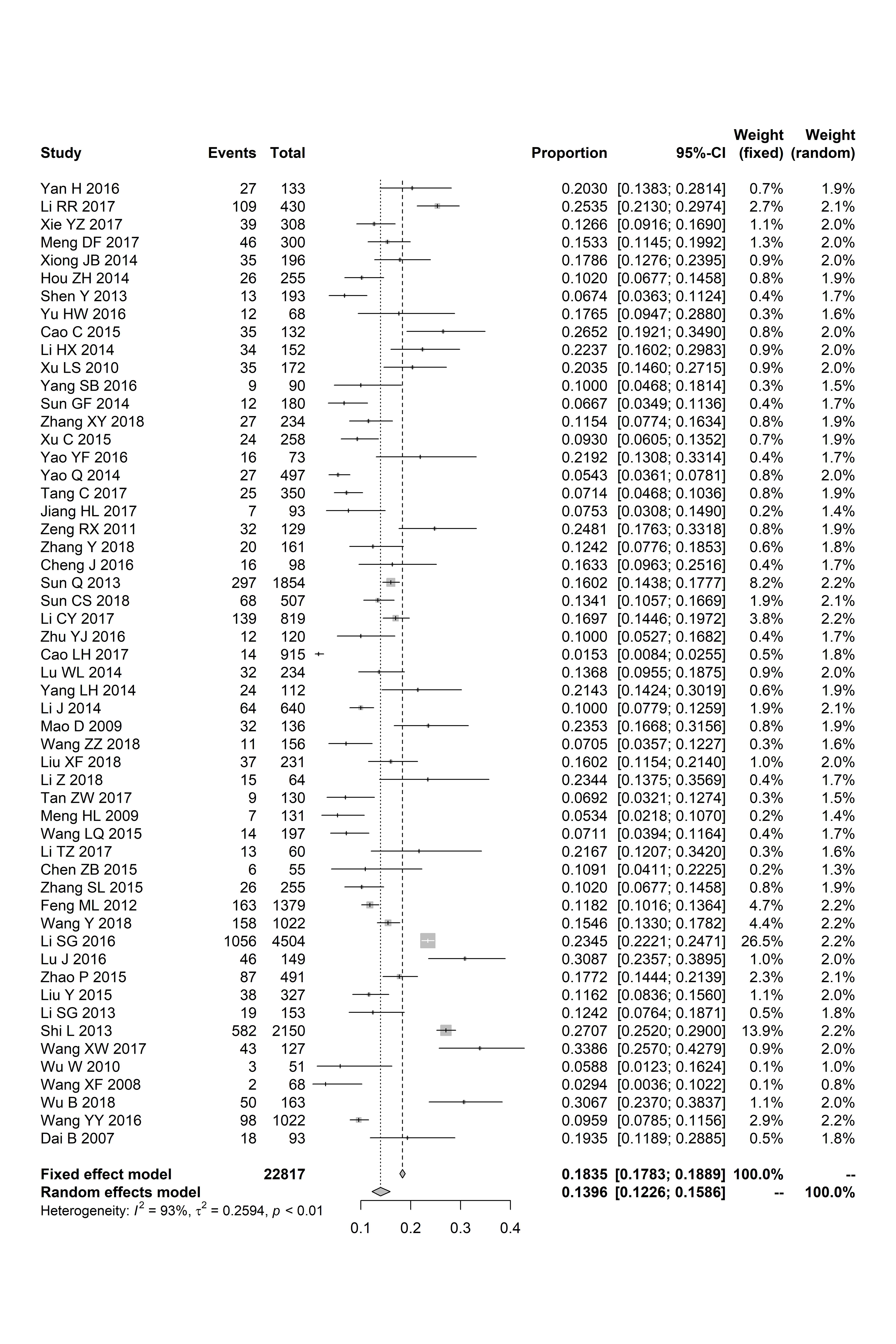

Supplement: Supplementary file 10 — High resolution image (TIF 7575 kb) [file 11657_2019_604_MOESM9_ESM.tif]

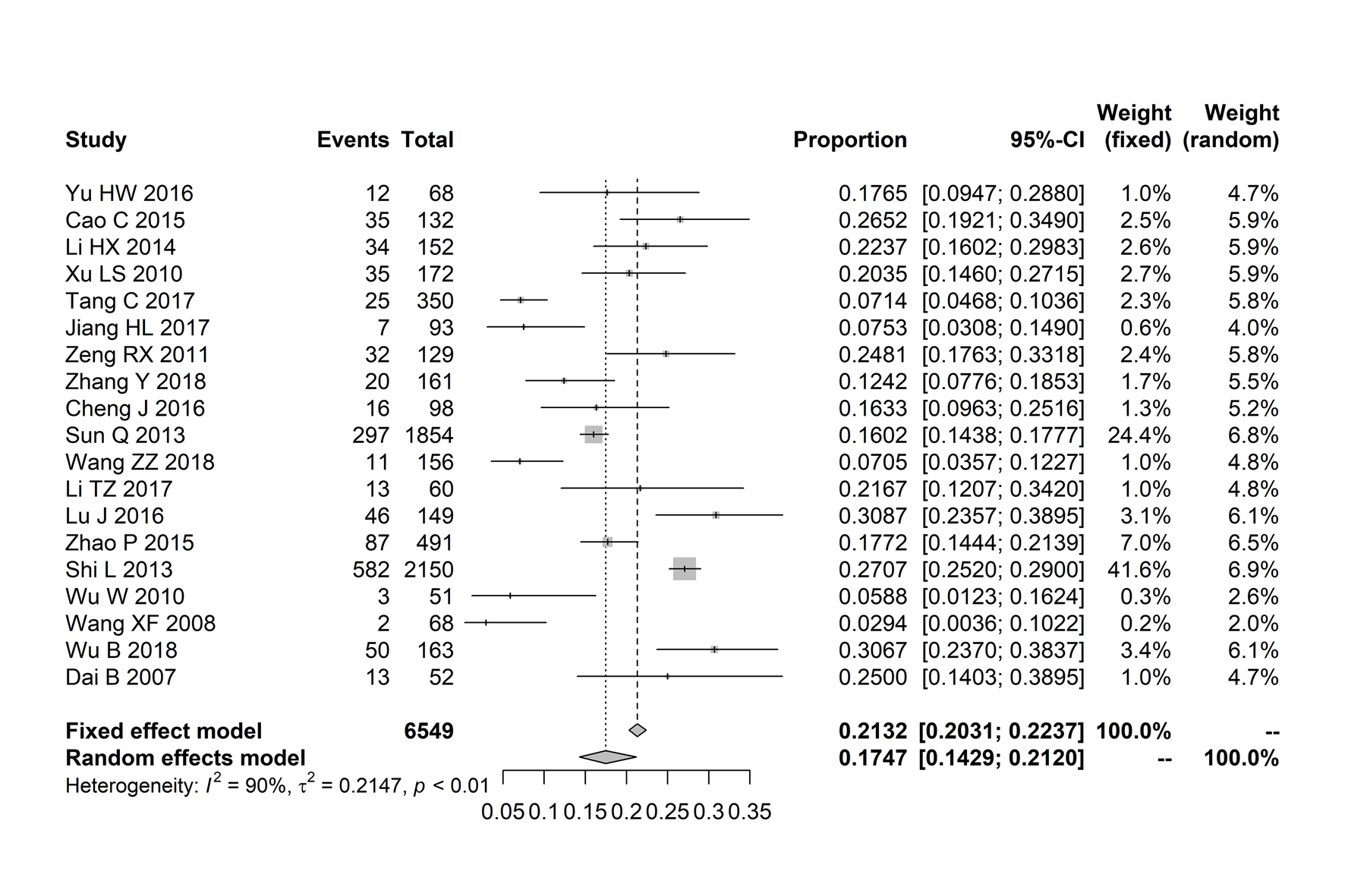

Supplement: Supplementary file 11 — Forest plot of the one-year mortality rate after femoral intertrochanteric fracture in mainland China. A total of 19 studies were included in the meta-analysis. The one-year mortality rate was calculated as 17.47% (95% CI 14.29% to 21.20%) using a random-effects model. (PNG 697 kb) [file 11657_2019_604_Fig6_ESM.png]

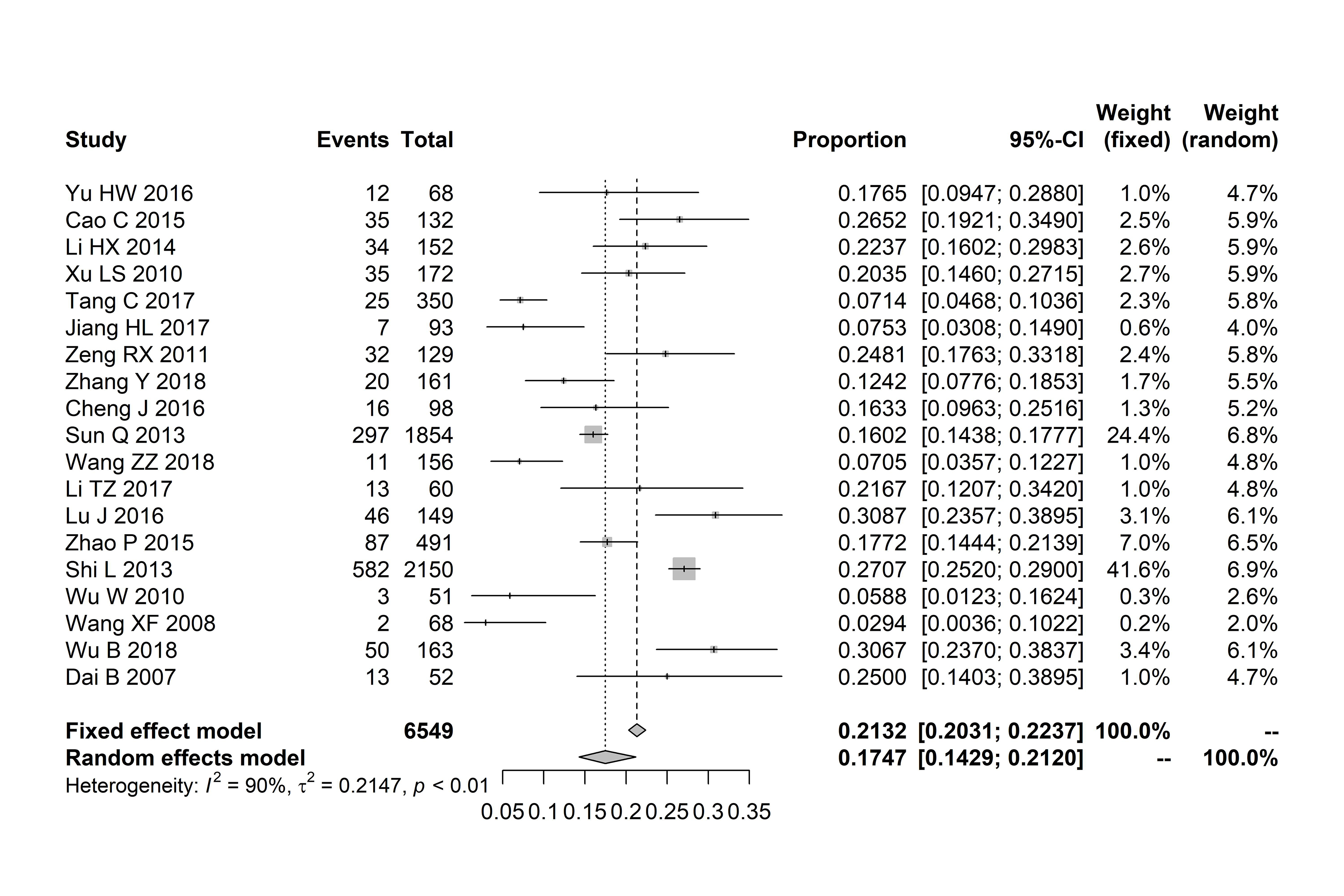

Supplement: Supplementary file 12 — High resolution image (TIF 3096 kb) [file 11657_2019_604_MOESM10_ESM.tif]

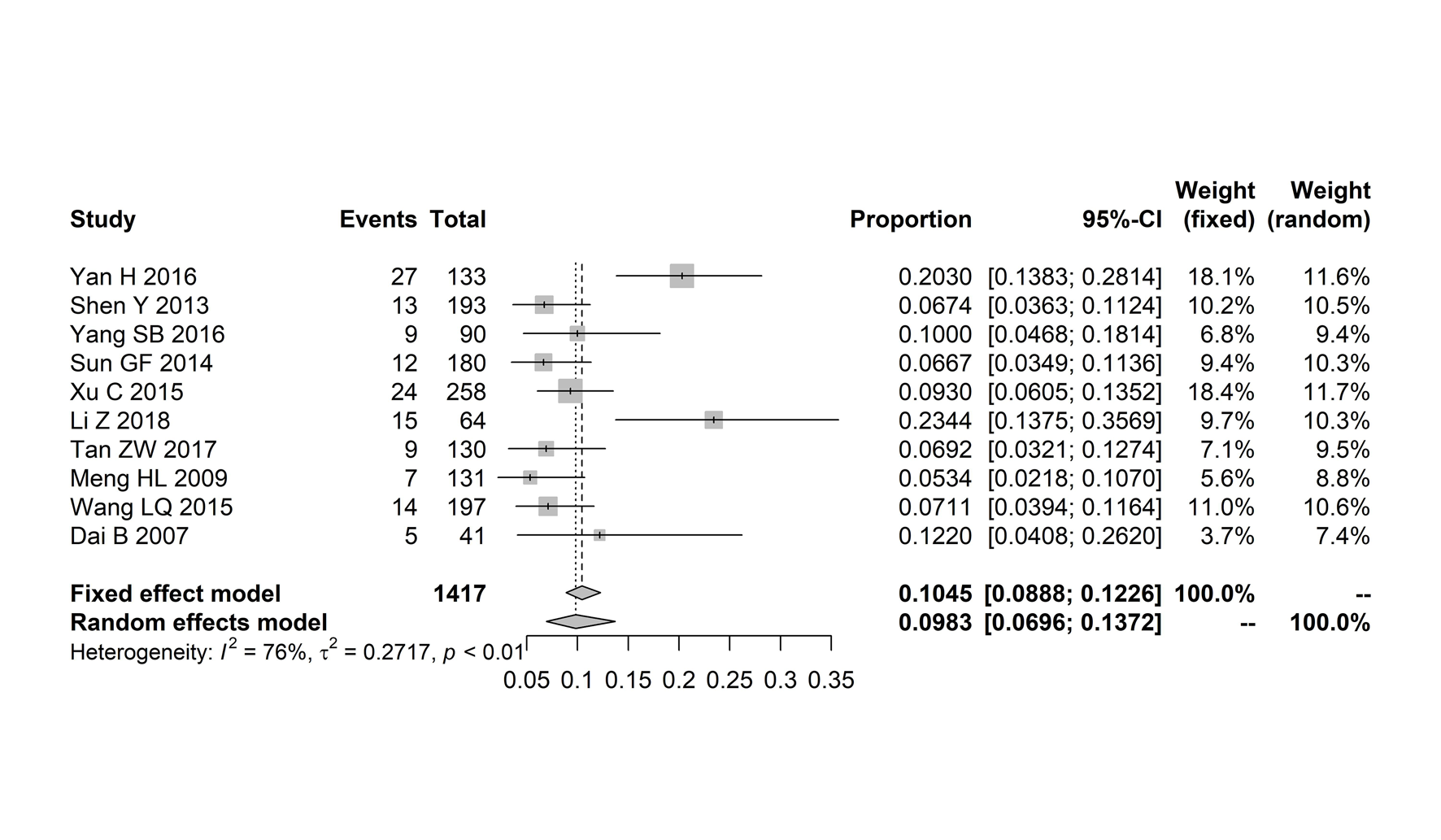

Supplement: Supplementary file 13 — Forest plot of the one-year mortality rate after femoral neck fracture in mainland China. A total of 10 studies were included in the meta-analysis. The one-year mortality rate was calculated as 9.83% (95% CI 6.96% to 13.72%) using a random-effects mode. (PNG 450 kb) [file 11657_2019_604_Fig7_ESM.png]

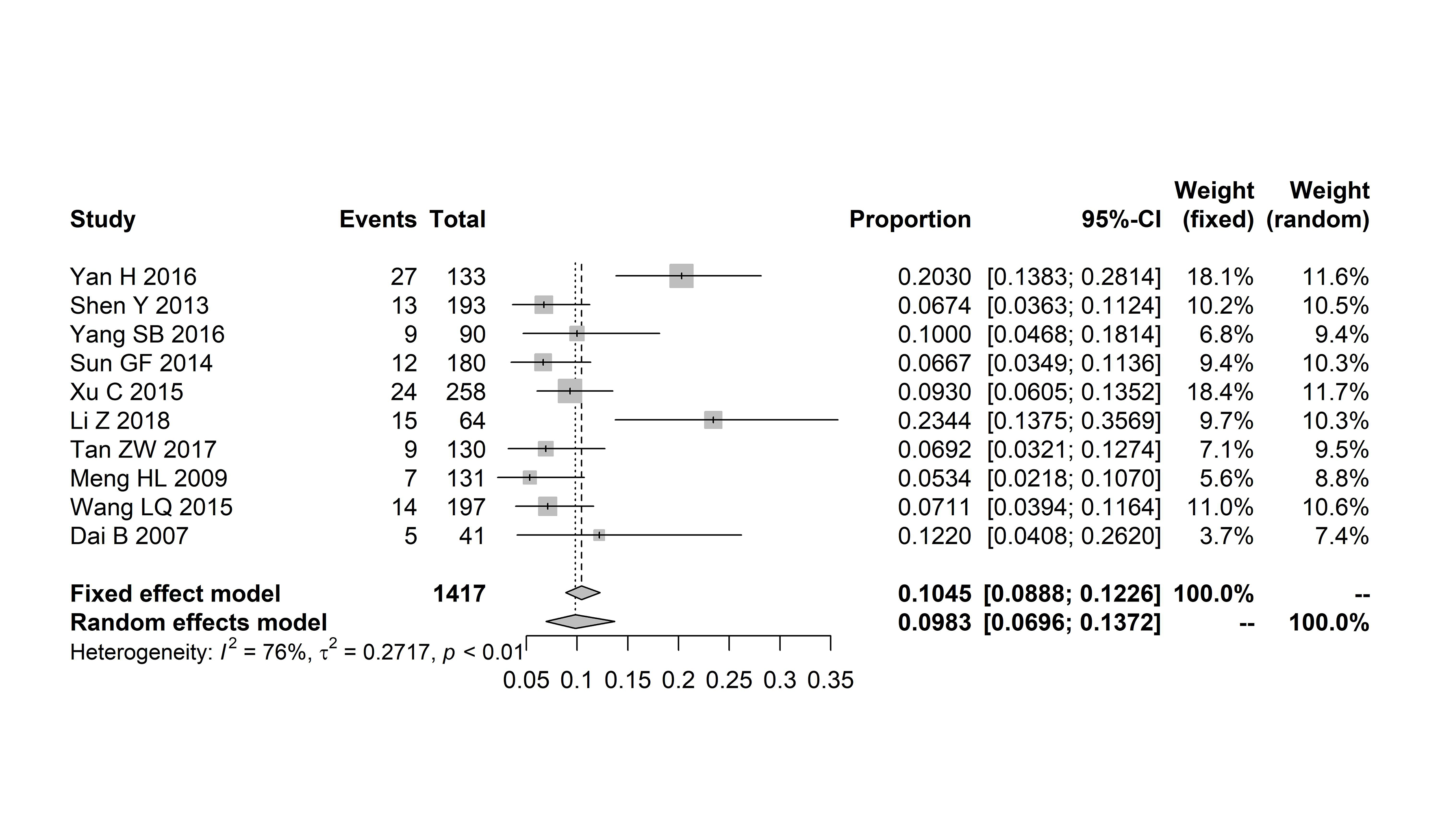

Supplement: Supplementary file 14 — High resolution image (TIF 1998 kb) [file 11657_2019_604_MOESM11_ESM.tif]

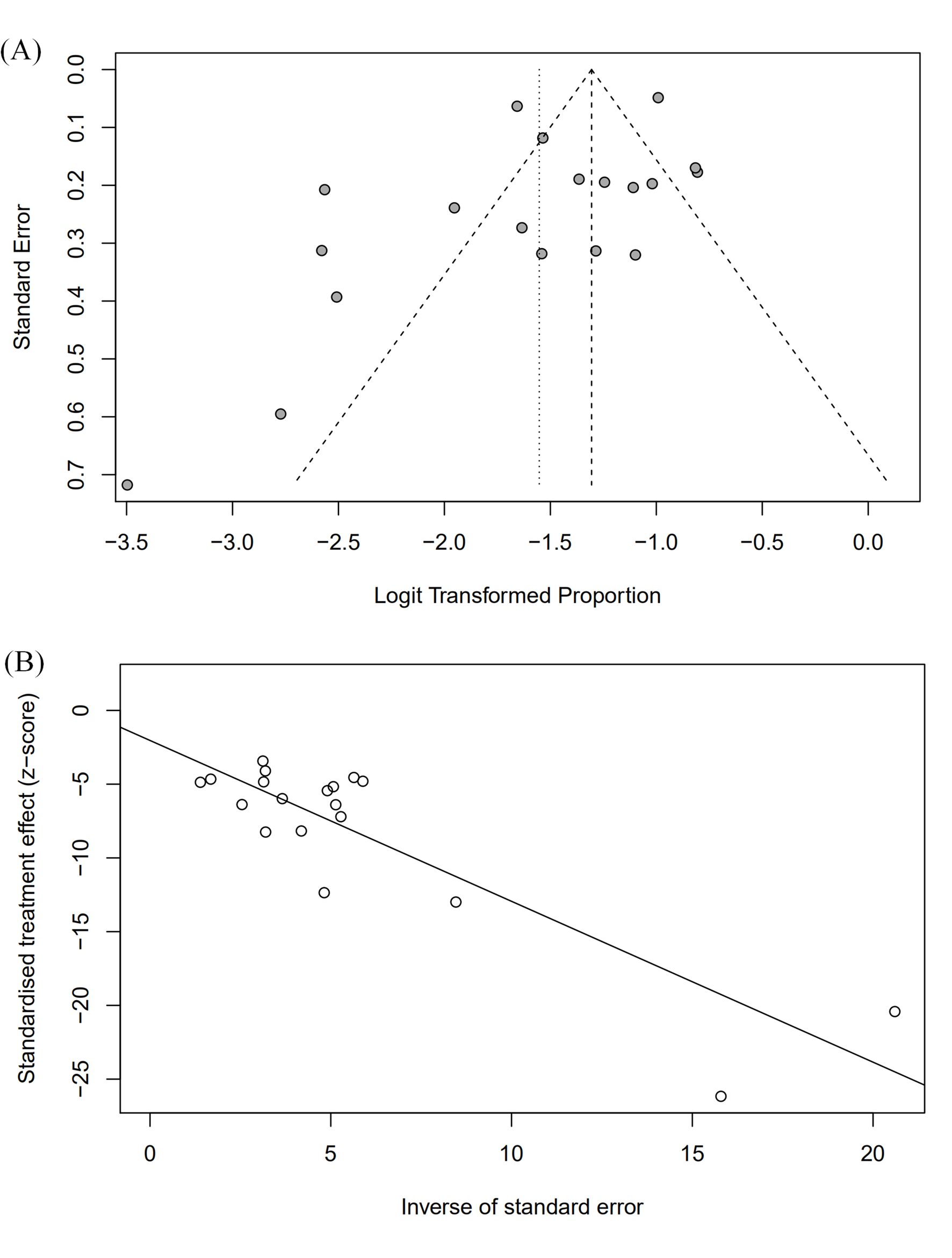

Supplement: Supplementary file 15 — Publication bias of one-year mortality after femoral intertrochanteric fracture. Note: (A) Funnel plot, (B) Egger’s test. (PNG 288 kb) [file 11657_2019_604_Fig8_ESM.png]

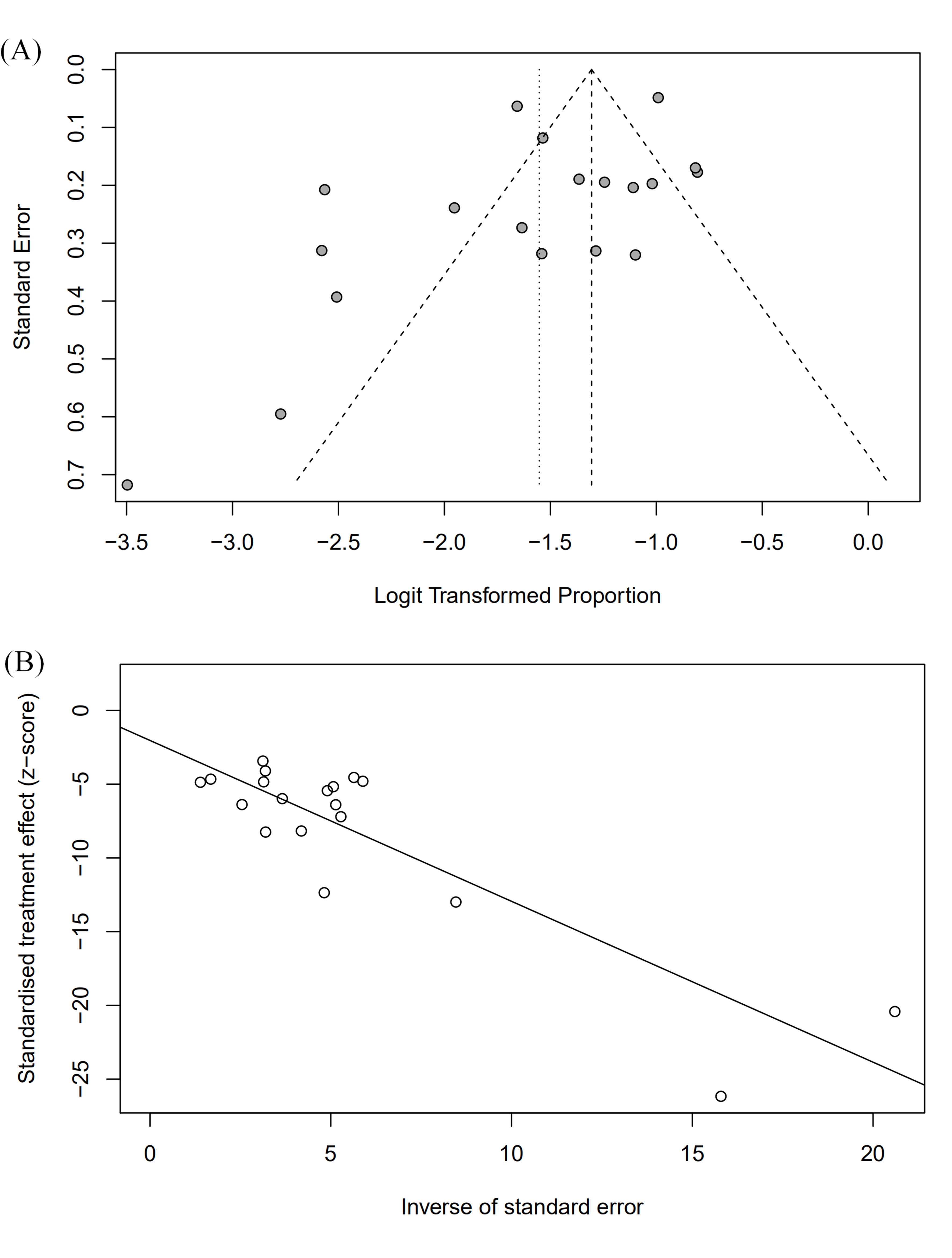

Supplement: Supplementary file 16 — High resolution image (TIF 3176 kb) [file 11657_2019_604_MOESM12_ESM.tif]

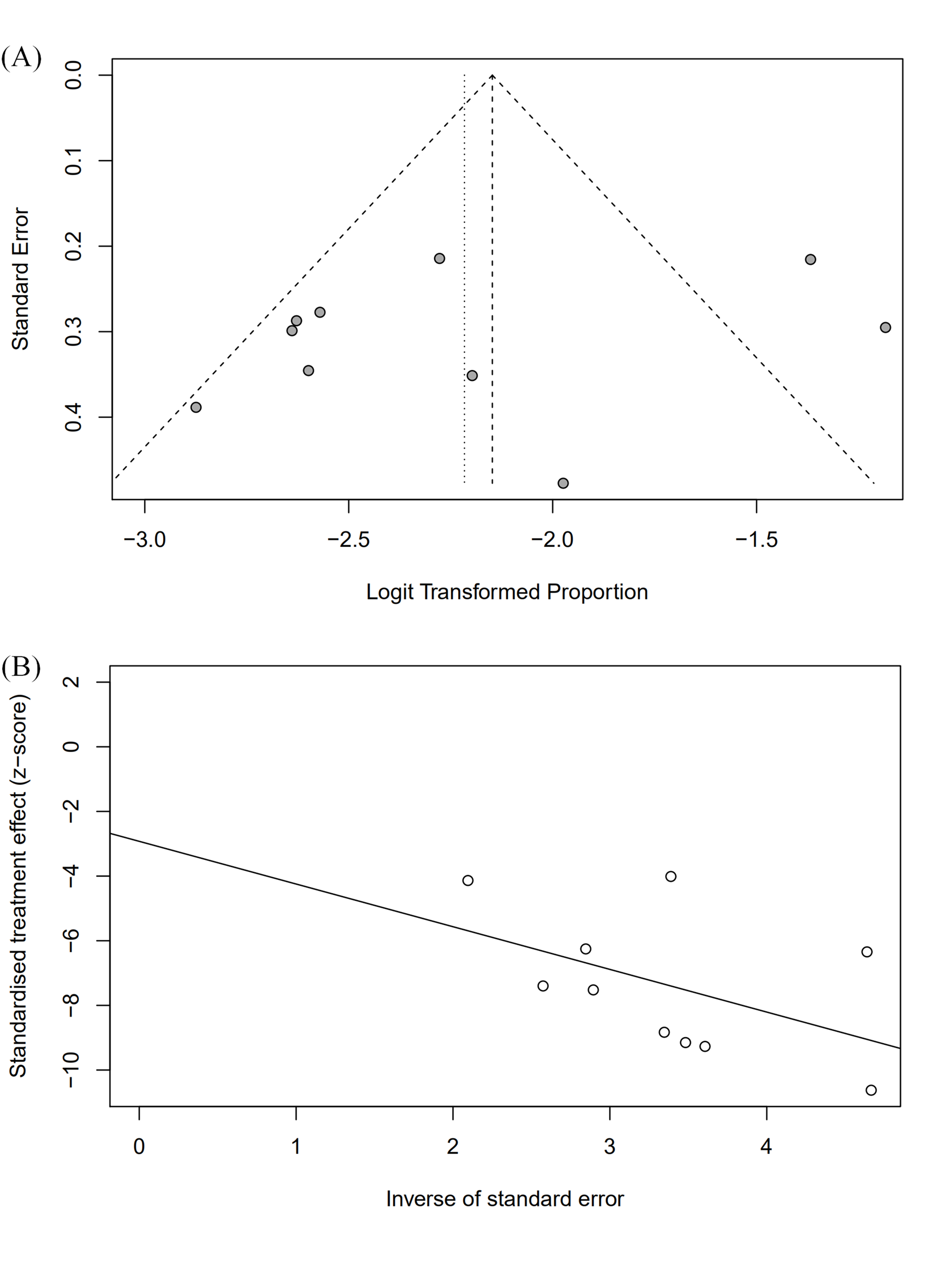

Supplement: Supplementary file 17 — Publication bias of one-year mortality after femoral neck fracture. Note: (A) Funnel plot, (B) Egger’s test. (PNG 239 kb) [file 11657_2019_604_Fig9_ESM.png]

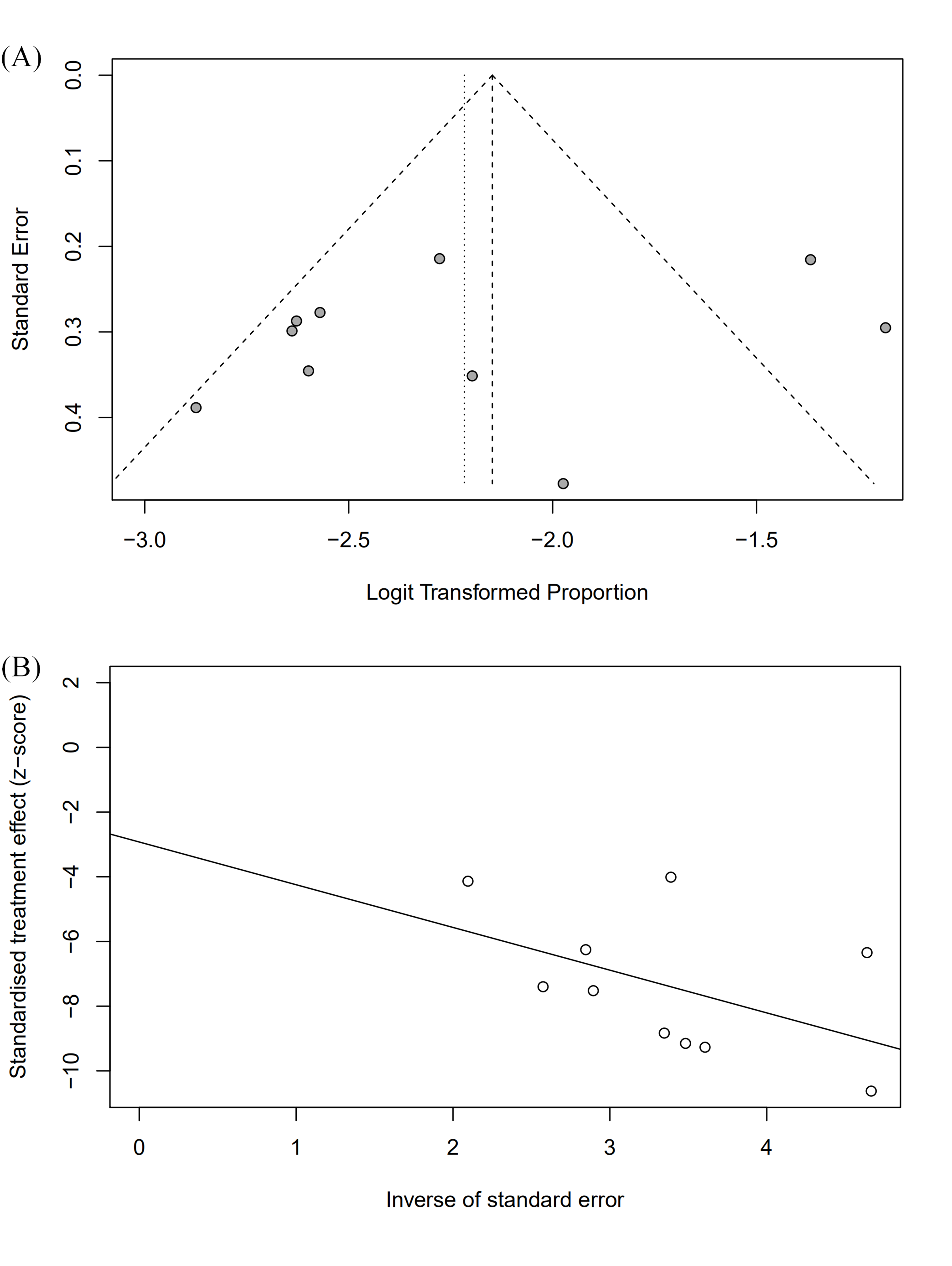

Supplement: Supplementary file 18 — High resolution image (TIF 2867 kb) [file 11657_2019_604_MOESM13_ESM.tif]

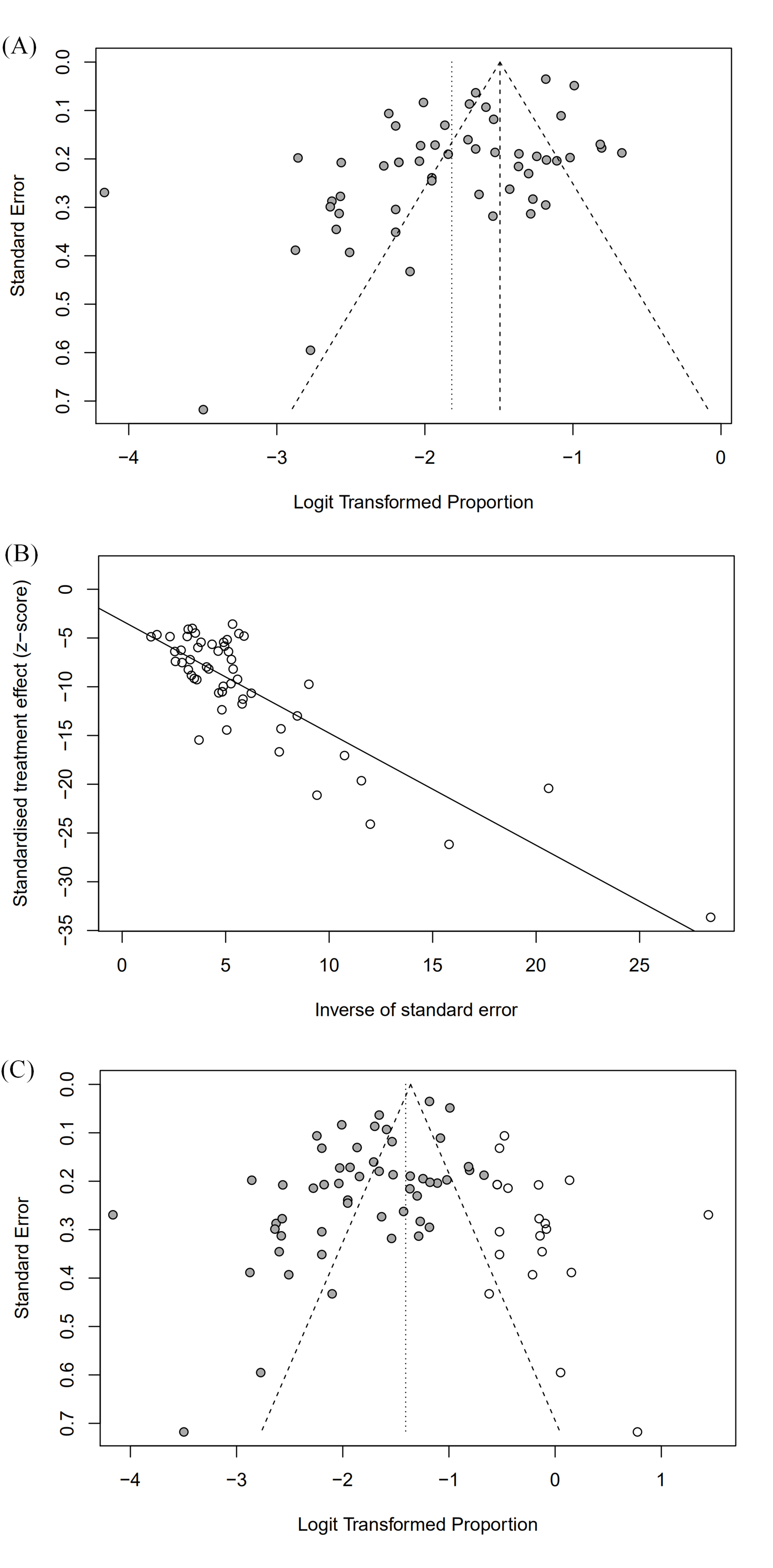

Supplement: Supplementary file 19 — Publication bias of one-year mortality after hip fracture. Note: (A) Funnel plot, (B) Egger’s test, (C) Funnel plot after the trim and fill method. (PNG 535 kb) [file 11657_2019_604_Fig10_ESM.png]

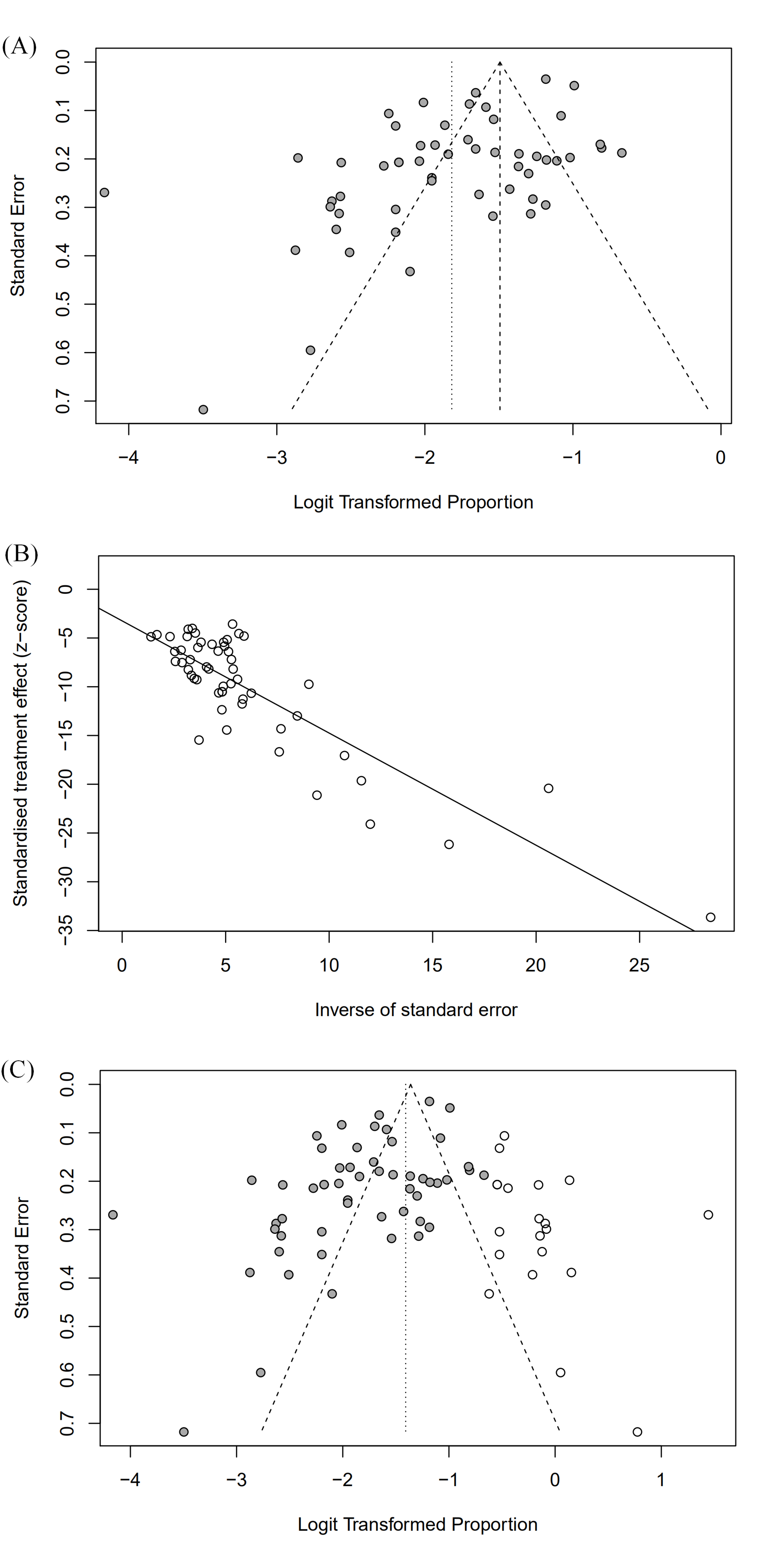

Supplement: Supplementary file 20 — High resolution image (TIF 5407 kb) [file 11657_2019_604_MOESM14_ESM.tif]

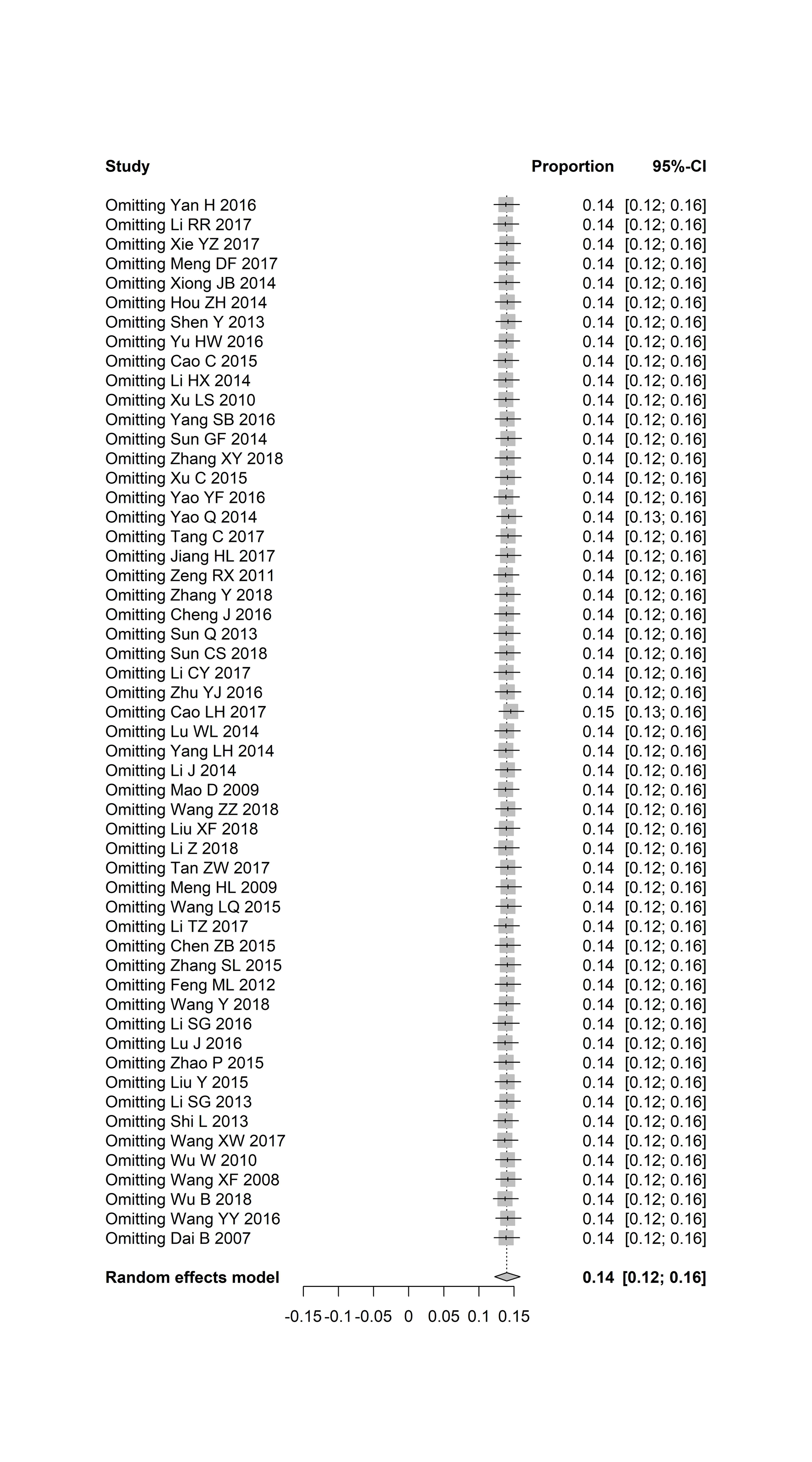

Supplement: Supplementary file 21 — Leave-one-out sensitivity analysis of one-year mortality after hip fracture. (PNG 1464 kb) [file 11657_2019_604_Fig11_ESM.png]

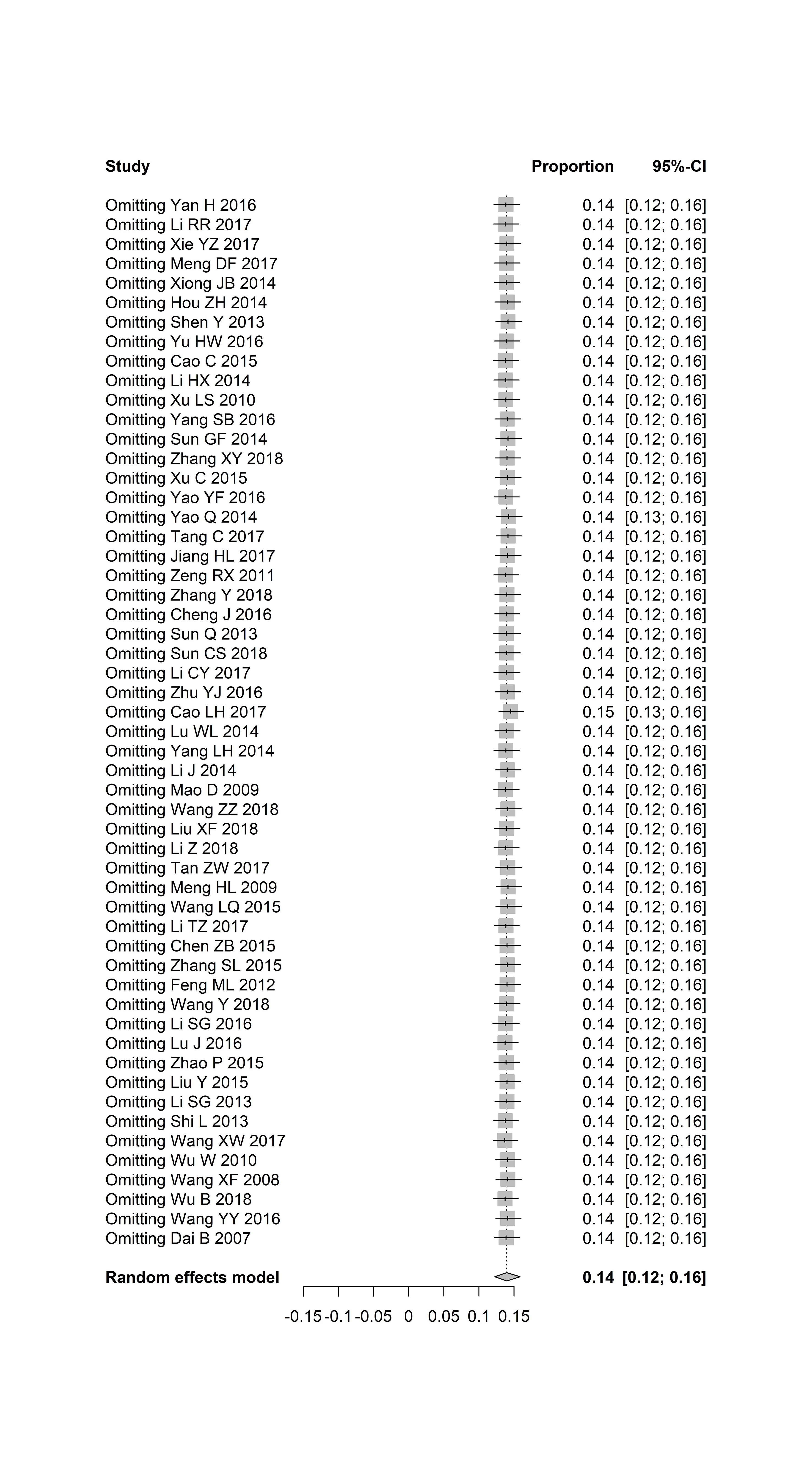

Supplement: Supplementary file 22 — High resolution image (TIF 7015 kb) [file 11657_2019_604_MOESM15_ESM.tif]

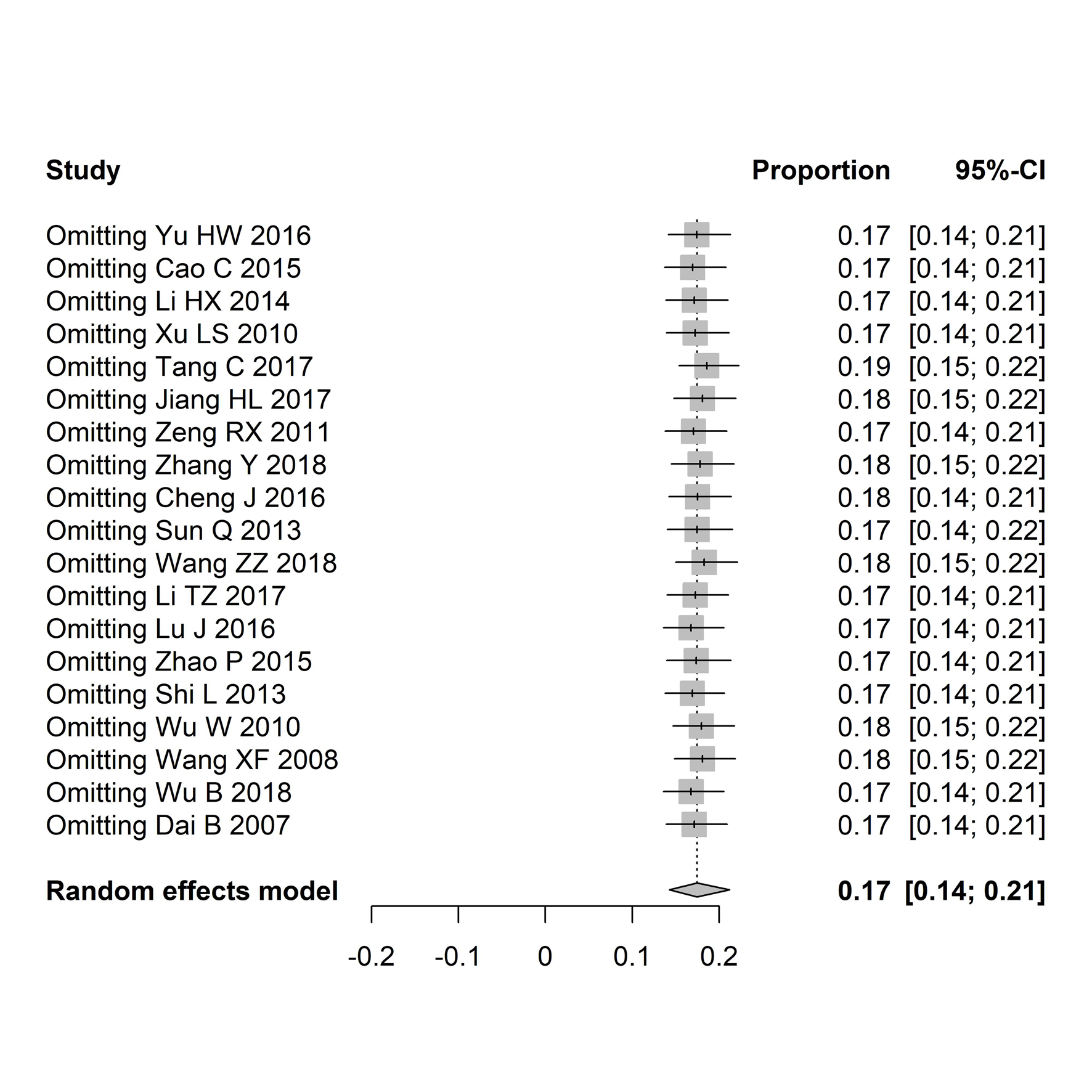

Supplement: Supplementary file 23 — Leave-one-out sensitivity analysis of one-year mortality after femoral intertrochanteric fracture. (PNG 788 kb) [file 11657_2019_604_Fig12_ESM.png]

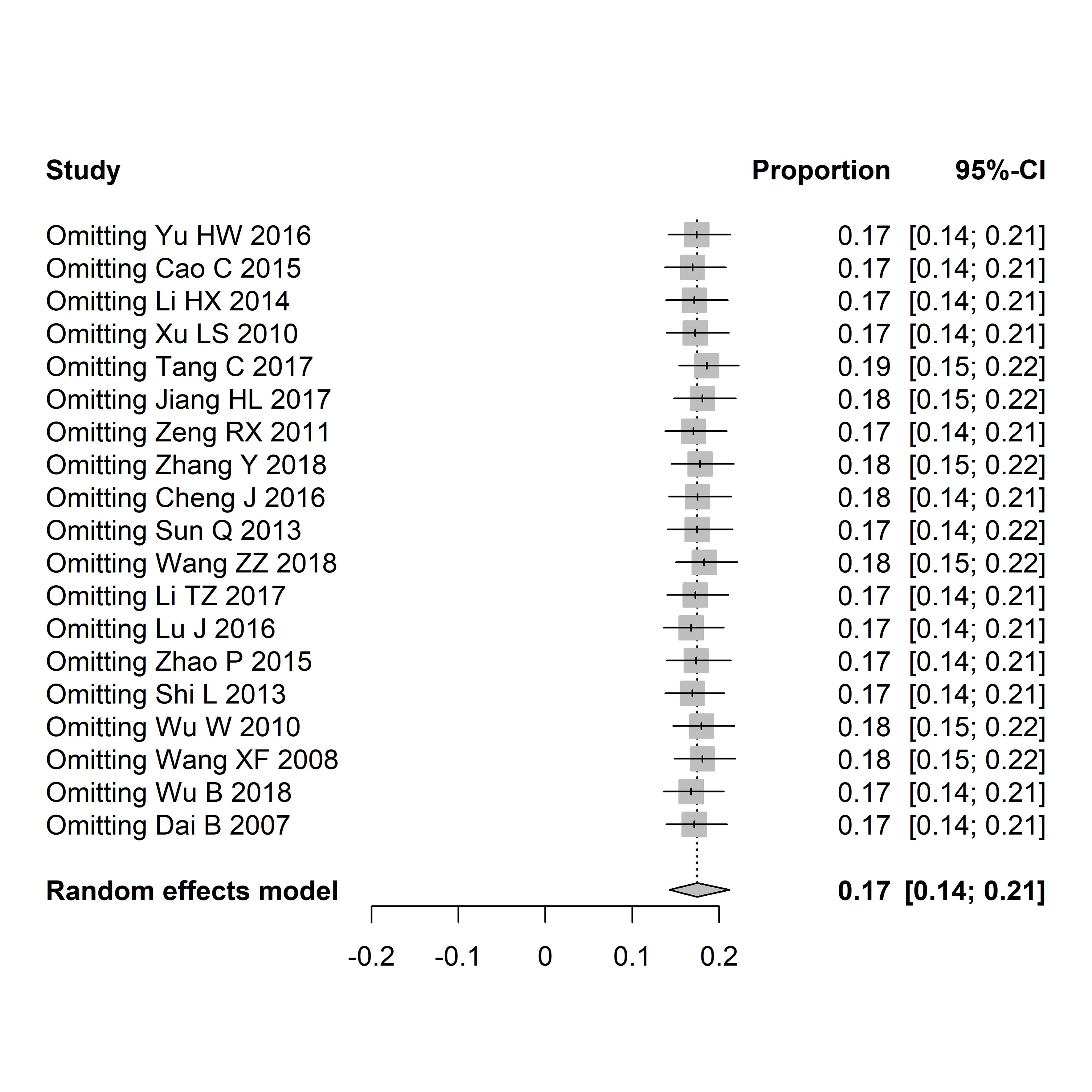

Supplement: Supplementary file 24 — High resolution image (TIF 3895 kb) [file 11657_2019_604_MOESM16_ESM.tif]

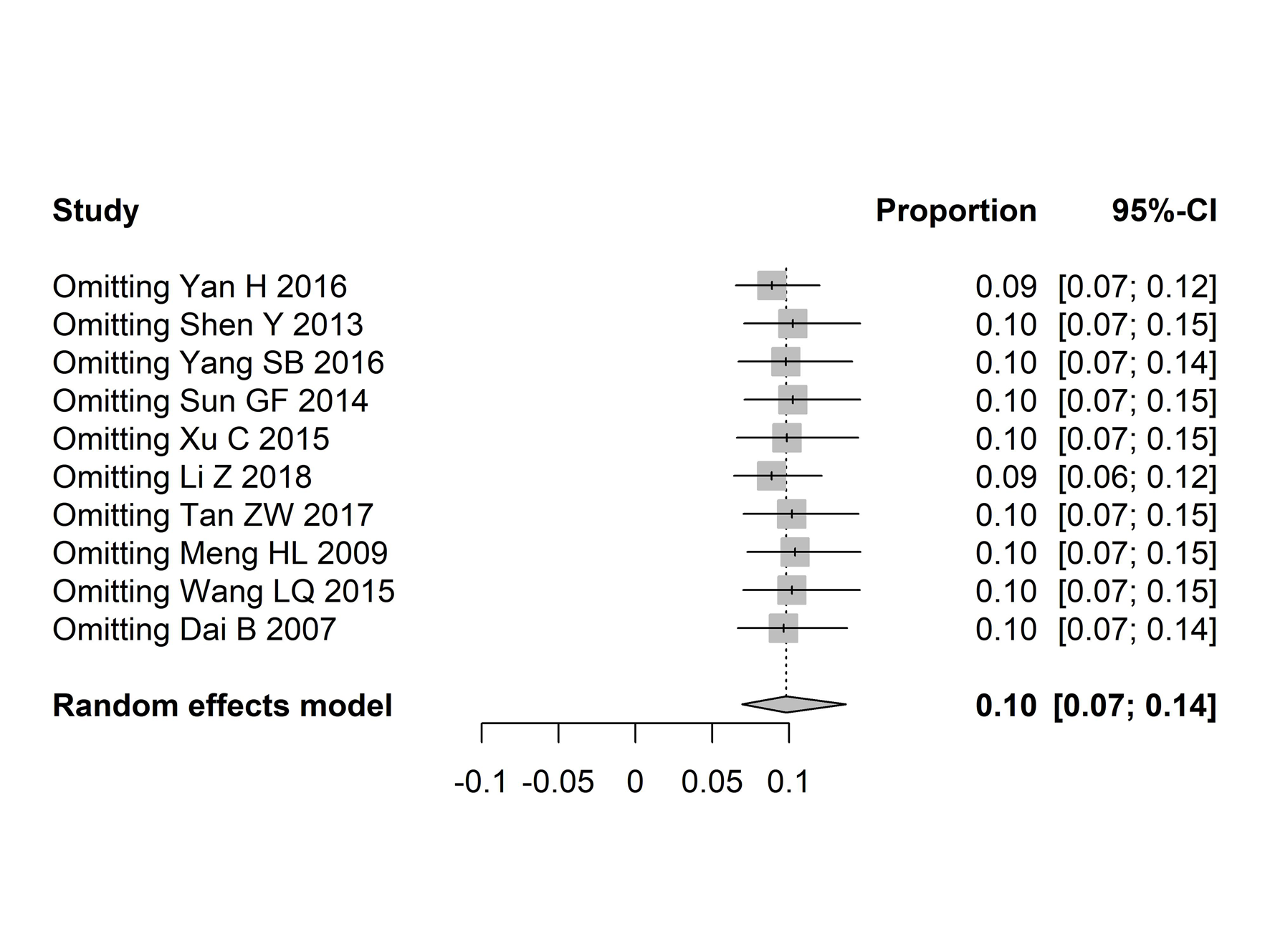

Supplement: Supplementary file 25 — Leave-one-out sensitivity analysis of one-year mortality after femoral neck fracture. (PNG 480 kb) [file 11657_2019_604_Fig13_ESM.png]

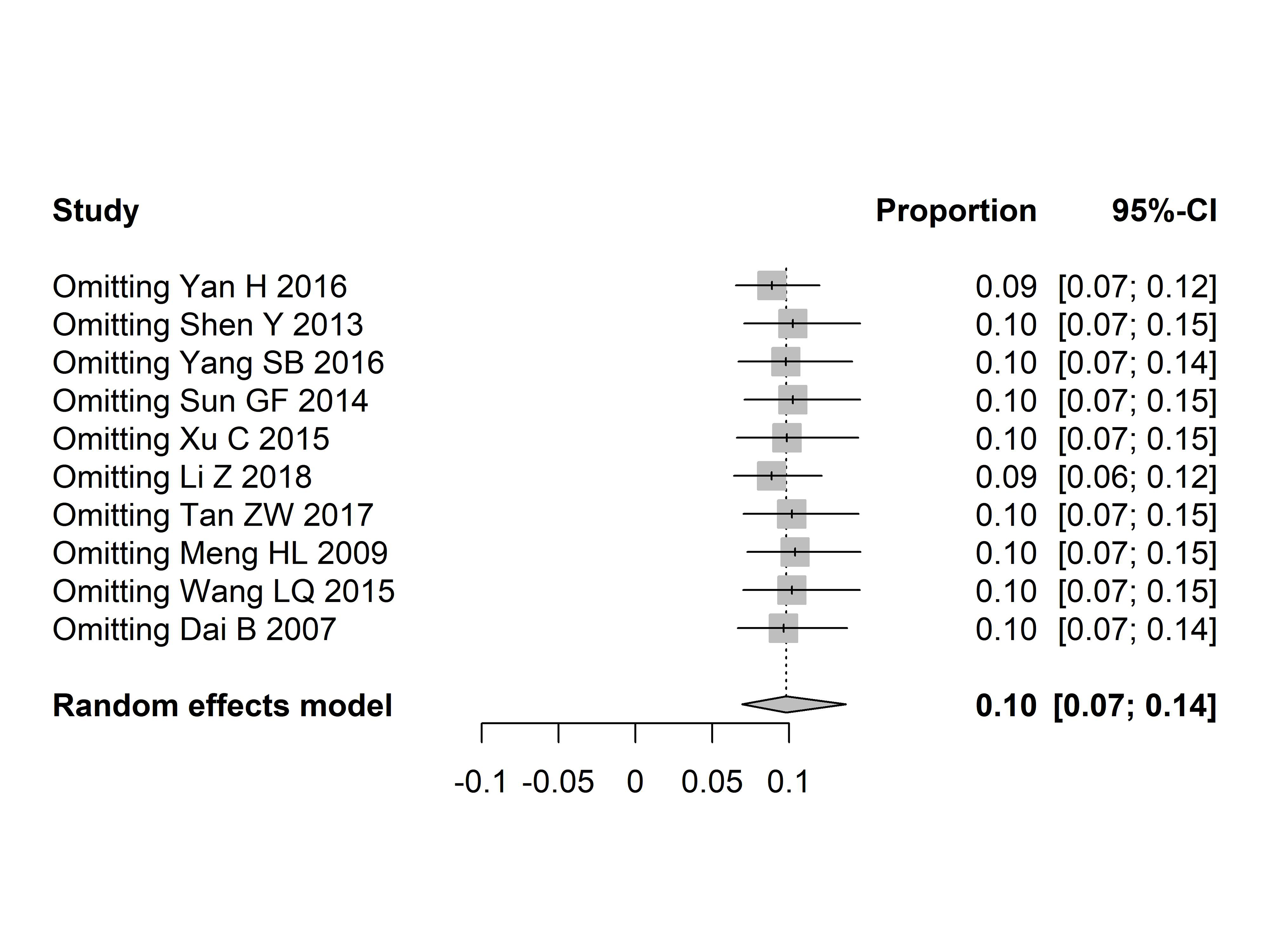

Supplement: Supplementary file 26 — High resolution image (TIF 2364 kb) [file 11657_2019_604_MOESM17_ESM.tif]
